# Reaction–Diffusion Problems on Time-Periodic Domains

**DOI:** 10.1007/s10884-023-10308-9

**Published:** 2023-09-23

**Authors:** Jane Allwright

**Affiliations:** Faculty of Science and Engineering, Swansea University, Singleton Park Campus, Swansea, SA2 8PP Wales, UK

**Keywords:** Time-periodic domain, Principal periodic eigenvalue, Reaction–diffusion

## Abstract

Reaction–diffusion equations are studied on bounded, time-periodic domains with zero Dirichlet boundary conditions. The long-time behaviour is shown to depend on the principal periodic eigenvalue of a transformed periodic-parabolic problem. We prove upper and lower bounds on this eigenvalue under a range of different assumptions on the domain, and apply them to examples. The principal eigenvalue is considered as a function of the frequency, and results are given regarding its behaviour in the small and large frequency limits. A monotonicity property with respect to frequency is also proven. A reaction–diffusion problem with a class of monostable nonlinearity is then studied on a periodic domain, and we prove convergence to either zero or a unique positive periodic solution.

## Introduction

In this paper we study reaction–diffusion equations on time-dependent domains $\Omega (t)\subset {\mathbb {R}}^N$ which are bounded, connected, and periodic with period *T*: $\Omega (t) \equiv \Omega (t+T)$. Specifically, we consider non-negative solutions $\psi (x,t)$ to

1 $$\begin{aligned} \frac{\partial \psi }{\partial t} = D \nabla ^2 \psi +f(\psi ) \qquad \text {for }x\in \Omega (t) \end{aligned}$$

2 $$\begin{aligned} \psi (x,t)=0 \qquad \text {for }x\in \partial \Omega (t) \end{aligned}$$

where *f* is either linear: $f(\psi )=f'(0)\psi$, or a nonlinear, Lipschitz continuous function which satisfies the following conditions for some $K>0$:

3 $$\begin{aligned}&f(0)=f(K)=0, \quad f'(0)>0\text { exists}, \quad \frac{f(k)}{k}\text { is non-increasing on }k>0. \end{aligned}$$

Note that these assumptions on *f* imply that $f(k)\le f'(0)k$ for $k> 0$.

Such reaction–diffusion problems, with $\psi \ge 0$, can be used to model population dynamics, chemical diffusion, and other biological, ecological and physical processes. We are interested in domains whose boundaries change periodically due to some external influence. In the context of population dynamics, this could represent a habitat whose boundary moves periodically due to the seasonal variation in temperature, water level, or snow cover, or due to a periodic cycle of land usage. Population models typically include Dirichlet, Neumann or Robin boundary conditions. In this study we shall impose zero Dirichlet conditions, since these lead to the more interesting mathematical results and comparison with the solution on a fixed domain.

For non-negative solutions to the problem (1), (2) but on a constant bounded domain $\Omega$, the long-time behaviour depends on $\lambda (\Omega )$, where this is the principal Dirichlet eigenvalue of $-\nabla ^2$ on $\Omega$. If $f'(0)<D\lambda (\Omega )$ then the solution tends to zero as $t\rightarrow \infty$. If $f'(0)>D\lambda (\Omega )$ then the solution to the linear equation tends to infinity, and the solution to the nonlinear problem has a nontrivial lower bound. (This is straightforward to show if we bound the initial conditions above and below by appropriately large and small multiples of the principal eigenfunction.) For an interval $0<x<L_0$ the critical threshold value for $f'(0)$ is $\frac{D\pi ^2}{L_0^2}$, which leads to the definition of ‘critical length’ as

$$\begin{aligned} L_{crit}=\pi \sqrt{\frac{D}{f'(0)}}. \end{aligned}$$

Thus, on a fixed interval $0<x<L_0$, there is a positive stationary solution to the linear problem if $L_0=L_{crit}$; otherwise the solution tends to zero or infinity depending whether $L_0<L_{crit}$ or $L_0>L_{crit}$ respectively. Such results only apply for constant domains, not for those that vary with time.

There is very little published work addressing similar matters on time-dependent domains; however see [1, 2] which consider the linear version of (1), (2) on time-dependent intervals $A(t)<x<A(t)+L(t)$. The approach in both [1, 2] is based upon a change of variables onto a fixed domain, followed by other changes of variables which allow the construction of exact solutions, subsolutions and supersolutions. Here, similarly, we start in Sect. 2 by transforming the problem onto a fixed domain. Now, due to the assumed *T*-periodicity of the original domain, this converts the problem to a periodic-parabolic equation. Although periodic-parabolic problems have been studied by several authors, and we make use of results from Castro and Lazer [4], Hess [5], Peng and Zhao [11], and Liu et al. [9], none of these have worked specifically on time-periodic domains or the periodic-parabolic equations that arise from them.

The results of Castro and Lazer [4] show that the long-time behaviour of the solution to our transformed problem is determined by a principal periodic eigenvalue, $\mu$. It is worth emphasising the importance of [4, Theorem 1] (the existence and uniqueness of the principal periodic eigenvalue and eigenfunction) for our problem on a time-periodic domain. The principal periodic eigenvalue $\mu$ is an important threshold value, and its existence distinguishes the study of time-periodic domains from that of general time-dependent domains. Various results concerning the long-time behaviour of the solution can now be stated in terms of bounds on this eigenvalue.

In Sect. 3 we derive upper and lower bounds on the principal periodic eigenvalue $\mu$ associated with a time-periodic domain $\Omega (t)$, under a range of different assumptions on $\Omega (t)$. These bounds, and their derivations, give original and useful insight into how problems on time-periodic domains behave. We also apply these bounds to some illustrative examples, including the interval $0<x<L(t)$ with

4 $$\begin{aligned} L(t)=L_0(1+\varepsilon \sin (\omega t)), \end{aligned}$$

with $\omega >0$ and $0<\varepsilon <1$. In this case (see Example 3), the bounds on $\mu$ imply that if

$$\begin{aligned} f'(0)<\frac{D\pi ^2}{L_0^2(1-\varepsilon ^2)^{3/2}}, \end{aligned}$$

that is, if

5 $$\begin{aligned} L_0 <\pi \sqrt{\frac{D}{f'(0)(1-\varepsilon ^2)^{3/2}}} = \frac{L_{crit}}{(1-\varepsilon ^2)^{3/4}}, \end{aligned}$$

then the solution to (1), (2) tends to zero. This result holds independently of the frequency $\omega >0$. Results from Sect. 4 also imply that if we have the opposite inequality,

6 $$\begin{aligned} L_0 > \frac{L_{crit}}{(1-\varepsilon ^2)^{3/4}}, \end{aligned}$$

then there exist $\omega >0$ such that the solution (to the linear version of (1), (2)) tends to infinity.

Such results demonstrate that the ‘critical length’ has a more intricate role for time-dependent domains than for constant domains. This is also shown in [2], where the author studies the problem on an interval $0<x<L(t)$ of general time-dependent length *L*(*t*). Even if $L(t)<L_{crit}$ for all *t*, the solution may or may not converge to zero. In [2], conditions on *L*(*t*) are derived that guarantee each outcome. In particular, if

7 $$\begin{aligned} L(t)=L_{crit}(1-\varepsilon (t+1)^{-k}) \end{aligned}$$

with $0<\varepsilon <1$ and $0<k\le 1$, then $\psi (x,t)\rightarrow 0$ uniformly in *x* as $t\rightarrow \infty$; however if $k>1$ then there is a non-trivial lower bound: $\psi (x,t)\ge B\sin \left( \frac{\pi x}{L(t)}\right)$ for some $B>0$.

Here, the case (4) is just one example that we consider; we also study more general time-periodic intervals $A(t)<x<A(t)+L(t)$. Moreover, several of our results are valid for time-periodic domains $\Omega (t)$ in ${\mathbb {R}}^N$.

In Sect. 4 we consider $\mu$ as a function of the frequency $\omega =\frac{2\pi }{T}$ and prove results concerning the small and large frequency limits, as well as a monotonicity property. The first step is to convert our principal eigenvalue problem into a 1-periodic problem; it then becomes an equation of the form

$$\begin{aligned} \frac{\omega }{2\pi }\frac{\partial \phi }{\partial s}- {\mathcal {L}}_{\omega }(\xi ,s)\phi = \mu (\omega )\phi (\xi ,s) \qquad \xi \in \Omega _0, s\in [0,1]. \end{aligned}$$

The operator ${\mathcal {L}}_{\omega }(\xi ,s)$ is 1-periodic in *s*, but it has certain terms that depend on, and scale with, $\omega$.

Our proofs of the limit $\lim _{\omega \rightarrow 0} \mu (\omega )$, and of a monotonicity result, are inspired by methods from [9] but have to be adapted to our own case. In [9], Liu et al. study the dependence of a principal periodic eigenvalue on the frequency, but for a different problem. They consider the principal periodic eigenvalue ${\hat{\lambda }}(\omega )$ of

$$\begin{aligned} \frac{\omega }{2\pi }\frac{\partial {\hat{\phi }}}{\partial s}- \mathcal {{\hat{L}}}(\xi ,s){\hat{\phi }} = {\hat{\lambda }}(\omega ){\hat{\phi }}(\xi ,s) \qquad \xi \in \Omega _0, s\in [0,1] \end{aligned}$$

where the coefficients of the operator $\mathcal {{\hat{L}}}(\xi ,s)$ are 1-periodic in *s* and do not depend on $\omega$. The result of [9, Theorem 1.3(i)] is that

8 $$\begin{aligned} \lim _{\omega \rightarrow 0} {\hat{\lambda }}(\omega ) = \int _0^1 \lambda ^{0}(s)ds \end{aligned}$$

where for each $0\le s\le 1$, $\lambda ^{0}(s)$ is the principal Dirichlet eigenvalue of the elliptic operator $-\mathcal {{\hat{L}}}(\xi ,s)$ on $\Omega _0$. This result can be extended in a natural way to the operator $-{\mathcal {L}}_{\omega }(\xi ,s)$, since the $\omega$-dependence is sufficiently smooth and the uniform ellipticity condition holds independently of $\omega$ as $\omega \rightarrow 0$. Thus, by adapting the proof of [9, Theorem 1.3(i)], in Theorem 5 we identify $\lim _{\omega \rightarrow 0} \mu (\omega )$ for a $\frac{2\pi }{\omega }$-periodic domain in any dimension. In Corollary 1 we then show that if $\mu (\omega )$ is the principal periodic eigenvalue associated with $\Omega (t)={\tilde{\Omega }}\left( \frac{\omega t}{2\pi }\right)$, where ${\tilde{\Omega }}(s)$ is a bounded and 1-periodic domain, then

9 $$\begin{aligned} \lim _{\omega \rightarrow 0}\mu (\omega ) =\int _0^1 D\lambda ({\tilde{\Omega }}(s))ds. \end{aligned}$$

In this formula, for each $0\le s\le 1$, $\lambda ({\tilde{\Omega }}(s))$ denotes the principal Dirichlet eigenvalue of $-\nabla ^2$ on ${\tilde{\Omega }}(s)$.

Next we consider $\omega \rightarrow \infty$. The large frequency limit of ${\hat{\lambda }}(\omega )$ (the principal periodic eigenvalue of Liu et al. in [9]) is given in [9, Theorem 1.3(ii)]. By adapting an argument from [10, Theorem 3.10], they prove that $\lim _{\omega \rightarrow \infty } {\hat{\lambda }}(\omega ) = \lambda _{\infty }$ where $\lambda _{\infty }$ is the principal eigenvalue of the elliptic operator whose coefficients are equal to the time-averages of those of $-\mathcal {{\hat{L}}}(\xi ,s)$. However, neither this result nor the analysis of [10] applies in cases such as ours, when some of the coefficients depend on $\omega$ and become unbounded as $\omega \rightarrow \infty$.

In our case, as we shall see, the behaviour of $\mu (\omega )$ as $\omega \rightarrow \infty$ depends on the detail of the problem. We show that very different types of asymptotic behaviour of $\mu (\omega )$ are possible as $\omega \rightarrow \infty$. Indeed, let

$$\begin{aligned} L(t)=L_0 l\left( \frac{\omega t}{2\pi }\right) , \qquad A(t)=A_0 a\left( \frac{\omega t}{2\pi }\right) \end{aligned}$$

where $L_0>0$, $A_0\ge 0$, $\omega >0$, and where $l(\cdot )$ and $a(\cdot )$ are 1-periodic functions with $l\ge 1$. Let $\mu (\omega )$ be the principal periodic eigenvalue associated with the domain $(A(t),A(t)+L(t))$. We show that if $a(\cdot )$ is constant then $\mu (\omega )=O(1)$ as $\omega \rightarrow \infty$, but if $a(\cdot )$ is non-constant then there exist $C_1$, $C_2$ such that: if $\frac{A_0}{L_0}<C_1$ then $\mu (\omega )=O(1)$ as $\omega \rightarrow \infty$, and if $\frac{A_0}{L_0}>C_2$ then $\mu (\omega ) \rightarrow \infty$ at the rate $\omega ^2$ as $\omega \rightarrow \infty$ (see Theorem 6).

In [9, Theorem 1.1], Liu et al. prove that if their parabolic operator has no advection term then the principal periodic eigenvalue ${\hat{\lambda }}(\omega )$ is non-decreasing with respect to $\omega >0$. In the final part of Sect. 4 we similarly show that the principal periodic eigenvalue $\mu (\omega )$ associated with a periodic domain $\Omega (t) \subset {\mathbb {R}}^N$ is also non-decreasing with respect to $\omega >0$.

We conclude the paper with Sect. 5, in which we consider the nonlinear problem on a periodic domain in ${\mathbb {R}}^N$, with *f* satisfying (3). Using a result of Hess [5] and methods involving the Poincaré map, we prove convergence to zero if $f'(0)<\mu$, or to a unique positive periodic solution if $f'(0)>\mu$. That is, if $f'(0)>\mu$ then there is a unique positive solution $\psi ^{*}(x,t)$ to (1), (2) such that $\psi ^{*}(x,t)\equiv \psi ^{*}(x,t+T)$, and for any non-negative initial conditions, the solution $\psi (x,nT+t)$ converges uniformly to $\psi ^{*}(x,t)$ as $n\rightarrow \infty$.

## Principal Periodic Eigenvalue $\mu$

Throughout the paper, we shall assume there is a one-to-one mapping $h(\cdot ,t): \overline{\Omega (t)}\rightarrow \overline{\Omega _0}$ which transforms $\Omega (t)$ into a bounded, connected reference domain $\Omega _0$ with sufficiently smooth boundary (at least $C^{2+\varepsilon }$ for some $\varepsilon >0$), and such that the change of variables $\xi =h(x,t)$ and $u(\xi ,t)=\psi (x,t)$ transforms (1), (2) into a parabolic equation of the form

10 $$\begin{aligned} \frac{\partial u}{\partial t} = {\mathcal {L}}(\xi ,t) u + f(u) \qquad \text {for } \xi \in \Omega _0 \end{aligned}$$

11 $$\begin{aligned} u(\xi ,t)=0\qquad \text {for } \xi \in \partial \Omega _0, \end{aligned}$$

where

12 $$\begin{aligned} {\mathcal {L}}(\xi ,t) u = \sum _{i,j}a_{ij}(\xi ,t)\frac{\partial ^2 u}{\partial \xi _i\partial \xi _j} +\sum _j \left( b_j(\xi ,t)+c_j(\xi ,t)\right) \frac{\partial u}{\partial \xi _j} \qquad \text {for } \xi \in \Omega _0, \end{aligned}$$

13 $$\begin{aligned} a_{ij}(\xi ,t)= \sum _{k}D\left( \frac{\partial h_i}{\partial x_k}\frac{\partial h_j}{\partial x_k}\right) , \qquad b_j(\xi ,t)= -\frac{\partial h_j}{\partial t}, \qquad c_j(\xi ,t)=D\nabla ^2 h_j. \end{aligned}$$

We assume that the map *h* is such that $a_{ij}$, $b_j$, $c_j$ belong to $C^{\alpha ,\alpha /2}(\overline{\Omega _0}\times [0,T])$ for some $\alpha >0$, and that $a_{ij}$ is uniformly elliptic. For example, if the time-dependent domain is an interval $A(t)<x<A(t)+L(t)$ where $L(t)>0$ and *A*(*t*) are $C^{2+\alpha }$ functions for some $\alpha >0$, then we can transform onto a fixed reference domain $0<\xi < L_0$ by letting $\xi =\left( \frac{x-A(t)}{L(t)}\right) L_0$. The solution $u(\xi ,t)=\psi (x,t)$ then satisfies

14 $$\begin{aligned} \frac{\partial u}{\partial t} = D \frac{L_0^2}{L(t)^2} \frac{\partial ^2 u}{\partial \xi ^2}+\left( \frac{{\dot{A}}(t)L_0+\xi {\dot{L}}(t)}{L(t)}\right) \frac{\partial u}{\partial \xi } +f(u) \qquad \text {in } 0< \xi < L_0 \end{aligned}$$

with $u(\xi ,t)=0$ at $\xi =0$ and $\xi =L_0$.

Since $\Omega (t)$ is periodic with period *T*, the map *h* and the coefficients of ${\mathcal {L}}$ are also *T*-periodic in *t*. By Theorem 1 of Castro and Lazer [4] there exists a value $\mu$ and a function $\phi (\xi ,t)$ such that

15 $$\begin{aligned} \frac{\partial \phi }{\partial t} - {\mathcal {L}} \phi = \mu \phi \qquad \text {in } \xi \in \Omega _0,\ t\in {\mathbb {R}} \end{aligned}$$

16 $$\begin{aligned} \phi (\xi ,t)=0 \qquad \qquad \text {for } \xi \in \partial \Omega _0 \end{aligned}$$

17 $$\begin{aligned} \phi (\xi ,t)>0 \qquad \qquad \text {for } \xi \in \Omega _0 \end{aligned}$$

18 $$\begin{aligned} \phi (\xi ,t)\equiv \phi (\xi ,t+T). \end{aligned}$$

This function $\phi$ is unique up to scaling [4, Theorem 1], and is called the principal periodic eigenfunction, while $\mu$ is called the principal periodic eigenvalue. Here, we shall say that $\mu$ is ‘the principal periodic eigenvalue of $\Omega (t)$’ to mean that it is the principal periodic eigenvalue of (15), (16), (17), (18), when ${\mathcal {L}}$ is defined by (12), (13).

The function $u(\xi ,t)=\phi (x,t)e^{(f'(0)-\mu )t}$ is a solution to the linear reaction–diffusion equation (10), (11) with $f(u)=f'(0) u$. So, if the initial conditions satisfy $b\phi (\xi ,0)\le u(\xi ,0)\le a\phi (\xi ,0)$ for some $0<b\le a$, then by the comparison principle the solution to the linear equation satisfies

19 $$\begin{aligned} b\phi (\xi ,t)e^{(f'(0)-\mu )t}\le u(\xi ,t)\le a\phi (\xi ,t)e^{(f'(0)-\mu )t} \qquad \text {for all }t\ge 0. \end{aligned}$$

The principal periodic eigenvalue is therefore a threshold such that if $f'(0)>\mu$ then $u(\xi ,t)\rightarrow \infty$ as $t\rightarrow \infty$, whereas if $f'(0)<\mu$ then $u(\xi ,t)\rightarrow 0$. See also [3, page 192].

## Bounds on $\mu$ and Examples

In this section we prove some bounds on $\mu$, the principal periodic eigenvalue of a *T*-periodic domain $\Omega (t)$. First we give a lower bound, which is valid for time-dependent domains in any dimension.

### Theorem 1

Let $\Omega (t)$ be *T*-periodic and let $\mu$ be the principal periodic eigenvalue of $\Omega (t)$. At each fixed time $0\le t\le T$, let $\lambda (\Omega (t))$ be the principal eigenvalue of $-\nabla ^2$ on the domain $\Omega (t)$ with zero Dirichlet boundary conditions. Then

20 $$\begin{aligned} \mu \ge \frac{1}{T}\int _0^T D\lambda (\Omega (t)) dt. \end{aligned}$$

### Proof

Let $\psi$ be a solution to the problem (1), (2) with $f(\psi )=f'(0) \psi$, and define

21 $$\begin{aligned} E(t)=\frac{1}{2}\int _{\Omega (t)}\psi (x,t)^2 dx. \end{aligned}$$

We differentiate *E*(*t*), noting that the additional contribution from the moving boundary $\partial \Omega (t)$ vanishes due to the zero Dirichlet conditions. It follows from (1), (2) that

22 $$\begin{aligned} \frac{dE}{dt}=\int _{\Omega (t)}\psi (D\nabla ^2\psi +f'(0)\psi )dx = \int _{\Omega (t)}(-D\vert \nabla \psi \vert ^2 +f'(0)\psi ^2)dx. \end{aligned}$$

Then for each time *t*, we use Poincaré’s inequality to get

23 $$\begin{aligned} \frac{dE}{dt}&\le \left( -D\lambda (\Omega (t)) +f'(0)\right) \int _{\Omega (t)} \psi ^2 dx =2\left( f'(0)-D\lambda (\Omega (t))\right) E(t). \end{aligned}$$

So for $$t\ge 0$$,

24 $$\begin{aligned} 0\le E(t)&\le E(0)\exp {\left( 2\int _0^t \left( f'(0)-D\lambda (\Omega (\zeta )) \right) d\zeta \right) }\nonumber \\&= E(0)\exp {\left( 2\left( f'(0)t - \frac{t}{T}\int _0^T D \lambda (\Omega (\zeta ))d\zeta \right) +O(1) \right) } \quad \text {as } t\rightarrow \infty \end{aligned}$$

where in the last line we have used the fact that $\lambda (\Omega (t))$ is *T*-periodic. Therefore, if $f'(0)-\frac{1}{T}\int _0^T D \lambda (\Omega (\zeta ))d\zeta <0$ then (24) implies that $\int _{\Omega (t)}\psi (x,t)^2 dx \rightarrow 0$ as $t\rightarrow \infty$. Therefore, the condition $f'(0)-\frac{1}{T}\int _0^T D \lambda (\zeta )d\zeta <0$ must imply that $f'(0)<\mu$, and so the bound (20) is proved. $\square$

### Remark 1

If we were instead interested in the principal periodic eigenvalue $\mu _V$ of an operator $\frac{\partial }{\partial t}-D\nabla ^2+V(x,t)$ on $\Omega (t)$, where *V* was a continuous function on $\Omega (t)$ and periodic in *t* with the same period as the domain, then the same proof would show that

$$\begin{aligned} \mu \ge \frac{1}{T}\int _0^T \left( D\lambda (\Omega (t))-\max _y V(y,t) \right) dt. \end{aligned}$$

### Example 1

Let $\Omega (t)=(A(t), A(t)+L(t))$ where $L(t)>0$ and *A*(*t*) are *T*-periodic. By Theorem 1,

25 $$\begin{aligned} \mu \ge \frac{1}{T}\int _0^T \frac{D\pi ^2}{L(t)^2} dt. \end{aligned}$$

In particular, if $L(t)\equiv l>0$ is constant and *A*(*t*) is periodic then we have the lower bound $\mu \ge \frac{D\pi ^2}{l^2}$. This means that whenever the solution on the fixed interval (0, *l*) tends to zero (i.e. $f'(0)<\frac{D\pi ^2}{l^2}$), the solution on a periodic interval $(A(t),A(t)+l)$ also tends to zero (i.e. $f'(0)<\mu$) for every periodic function *A*(*t*).

The next result follows from the comparison principle, and is also valid for time-dependent domains in any dimension.

### Theorem 2

Let $\Omega (t)$ be *T*-periodic and let $\mu$ be the principal periodic eigenvalue of $\Omega (t)$. Suppose there is a domain $\Omega _1$ such that $\Omega _1 \subset \Omega (t)$ for all *t*, and let $\lambda (\Omega _1)$ be the principal eigenvalue of $-\nabla ^2$ on $\Omega _1$ with zero Dirichlet boundary conditions. Then

26 $$\begin{aligned} \mu \le D\lambda (\Omega _1). \end{aligned}$$

### Proof

Let $\psi _1$ be the solution to (1), (2) with $f(u)=f'(0) u$, but on the fixed domain $\Omega _1$, and with non-trivial initial conditions satisfying $0\le \psi _1(x,0)\le \psi (x,0)$. By the comparison principle, $0\le \psi _1(x,t)\le \psi (x,t)$ for all $x\in \Omega _1$ and $t\ge 0$. If $f'(0)<\mu$ then $\psi \rightarrow 0$ as $t\rightarrow \infty$, which implies that $\psi _1\rightarrow 0$ and so that $f'(0)<D\lambda (\Omega _1)$. Therefore we conclude that $\mu \le D\lambda (\Omega _1)$. $\square$

### Example 2

Let $\Omega (t)=(A(t), A(t)+L(t))$ for some *T*-periodic functions $L(t)>0$ and *A*(*t*) satisfying

27 $$\begin{aligned} \max _{[0,T]} A < \min _{[0,T]}(A+L). \end{aligned}$$

The fixed interval $\Omega _1:=(\max (A), \min (A+L))$ is always contained within the domain $(A(t), A(t)+L(t))$. By Theorem 2, we get the upper bound

28 $$\begin{aligned} \mu \le \frac{D\pi ^2}{(\min (A+L) - \max A)^2}. \end{aligned}$$

In particular, if $A(t)\equiv A(0)$ is constant and $L(t)>0$ is periodic, then $\mu \le \frac{D\pi ^2}{(\min L)^2}$.

Let us continue to consider to the linear equation on the time-dependent interval $A(t)<x<A(t)+L(t)$ where $L(t)>0$ and *A*(*t*) are *T*-periodic and belong to $C^{2+\alpha }([0,T])$ for some $\alpha >0$. We shall apply the same changes of variables that were introduced in [1, 2]. Namely, first we transform onto a fixed reference domain $0<\xi < L_0$ by letting $\xi =\left( \frac{x-A(t)}{L(t)}\right) L_0$. The solution $u(\xi ,t)=\psi (x,t)$ then satisfies Eq. (14) with $f(u)=f'(0)u$, and $u(\xi ,t)=0$ at $\xi =0$ and $\xi =L_0$. Next, we let $w(\xi ,t)=u(\xi ,t)H(\xi ,t)e^{-f'(0)t}$ where

29 $$\begin{aligned} H(\xi , t)=\left( \frac{L(t)}{L(0)}\right) ^{1/2}\exp {\left( \int \limits _0^t \frac{{\dot{A}}(\zeta )^2}{4D} d\zeta +\frac{\xi ^2 {\dot{L}}(t)L(t)}{4DL_0^2}+\frac{\xi {\dot{A}}(t)L(t)}{2DL_0}\right) }. \end{aligned}$$

As in [1, 2], we find that *w* satisfies the problem

30 $$\begin{aligned} \frac{\partial w}{\partial t} = D \frac{L_0^2}{L(t)^2} \frac{\partial ^2 w}{\partial \xi ^2}+\left( \frac{\xi ^2{\ddot{L}}(t)L(t)}{4DL_0^2} + \frac{\xi {\ddot{A}}(t)L(t)}{2DL_0} \right) w \qquad \text {in } 0< \xi < L_0 \end{aligned}$$

31 $$\begin{aligned} w(\xi ,t)=0 \qquad \text {\qquad }\text {at } \xi =0 \text { and } \xi =L_0. \end{aligned}$$

From [2] we have the following result.

### Theorem 3

[2, Theorem 2.1] Let $w(\xi ,t) \ge 0$ satisfy (30), (31), and assume that $C_1 \sin \left( \frac{\pi \xi }{L_0}\right) \le w(\xi ,0) \le C_2 \sin \left( \frac{\pi \xi }{L_0}\right)$ for some $0<C_1\le C_2$. Define

32 $$\begin{aligned} {\overline{Q}}(t)=\max _{0\le \eta \le 1} \left( \frac{\eta ^2{\ddot{L}}(t)L(t)}{2} + \eta {\ddot{A}}(t)L(t) \right) ,\nonumber \\ {\underline{Q}}(t)=-\min _{0\le \eta \le 1} \left( \frac{\eta ^2{\ddot{L}}(t)L(t)}{2} + \eta {\ddot{A}}(t)L(t) \right) . \end{aligned}$$

Then for every $t\ge 0$,

33 $$\begin{aligned} C_1 \sin \left( \frac{\pi \xi }{L_0}\right) e^{\int _0^t \left( -\frac{D\pi ^2}{L(\zeta )^2}-\frac{{\underline{Q}}(\zeta )}{2D}\right) d\zeta }\le w(\xi ,t)\le C_2\sin \left( \frac{\pi \xi }{L_0}\right) e^{\int _0^t \left( -\frac{D\pi ^2}{L(\zeta )^2}+\frac{{\overline{Q}}(\zeta )}{2D}\right) d\zeta }. \end{aligned}$$

### Proof

By the definitions of ${\overline{Q}}(t)$ and ${\underline{Q}}(t)$, and the Eq. (30) satisfied by $w(\xi ,t)\ge 0$, we have

34 $$\begin{aligned} -\frac{{\underline{Q}}(t)}{2D}w(\xi ,t)\le \frac{\partial w}{\partial t}-D\frac{L_0^2}{L(t)^2}\frac{\partial ^2 w}{\partial \xi ^2} \le \frac{{\overline{Q}}(t)}{2D}w(\xi ,t) \qquad \text {in } 0<\xi <L_0. \end{aligned}$$

By applying the parabolic comparison principle on $[0,L_0]\times [0,t]$, we obtain the lower and upper bounds stated. $\square$

Let $\mu _u=\mu$ denote the principal periodic eigenvalue of the operator which acts on *u* in Eq. (14), and let $\mu _w$ denote the principal periodic eigenvalue of the operator which acts on *w* in Eq. (30). As in Eq. (19) we know that

35 $$\begin{aligned} u(\xi ,t)=\overline{{\underline{O}}} (\phi _u(\xi ,t)e^{(f'(0)-\mu _u)t}), \qquad w(\xi ,t)=\overline{{\underline{O}}} (\phi _w(\xi ,t)e^{-\mu _w t}) \end{aligned}$$

where $\phi _u(\xi ,t)$ and $\phi _w(\xi ,t)$ are the principal periodic eigenfunctions associated with $\mu _u$ and $\mu _w$, and where we use the notation $u_1=\overline{{\underline{O}}}(u_2)$ to mean that $u_1=O(u_2)$ and $u_2=O(u_1)$. The relationship between $\mu _u$ and $\mu _w$ is given in the following lemma.

### Lemma 1

Let $\mu _u=\mu$ denote the principal periodic eigenvalue of the operator which acts on *u* in Eq. (14), and let $\mu _w$ denote the principal periodic eigenvalue of the operator which acts on *w* in Eq. (30) . Then

36 $$\begin{aligned} \mu _u = \mu _w+\frac{1}{T}\int _0^T \frac{{\dot{A}}(t)^2}{4D}dt. \end{aligned}$$

### Proof

Consider the function $H(\xi ,t)$ given by (29), which occurs in the change of variables from *u* to *w*. Since $L(t)>0$ and *A*(*t*) are both periodic, note that

37 $$\begin{aligned} \left( \frac{L(t)}{L(0)}\right) ^{1/2}\exp {\left( \frac{\xi ^2 {\dot{L}}(t)L(t)}{4DL_0^2}+\frac{\xi {\dot{A}}(t)L(t)}{2DL_0}\right) } = \overline{{\underline{O}}}(1) \end{aligned}$$

in the sense that the left-hand side has a finite upper bound a positive lower bound, uniformly in $t\ge 0$, $0\le \xi \le L_0$. Therefore from the change of variables and the periodicity of ${\dot{A}}(t)$, we have

38 $$\begin{aligned} u(\xi ,t)=&\overline{{\underline{O}}}\left( w(\xi ,t)e^{f'(0)t}\exp \left( -\int \limits _0^t \frac{{\dot{A}}(\zeta )^2}{4D} d\zeta \right) \right) \nonumber \\ =&\overline{{\underline{O}}}\left( w(\xi ,t)\exp \left( f'(0)t -\frac{t}{T} \int \limits _0^T \frac{{\dot{A}}(\zeta )^2}{4D} d\zeta \right) \right) . \end{aligned}$$

The claimed relationship (36) then follows by combining (38) with (35). $\square$

We also derive upper and lower bounds on $\mu _w$ and hence, via Eq. (36), upper and lower bounds on $\mu _u$.

### Theorem 4

Let $\mu =\mu _u$ denote the principal periodic eigenvalue of the operator which acts on *u* in Eq. (14). Let ${\overline{Q}}(t)$, ${\underline{Q}}(t)$ be given by Eq. (32). Then

39 $$\begin{aligned} \frac{1}{T}\int _0^T \left( \frac{D\pi ^2}{L(t)^2} +\frac{{\dot{A}}(t)^2}{4D} -\frac{{\overline{Q}}(t)}{2D}\right) dt \le \mu \le \frac{1}{T}\int _0^T \left( \frac{D\pi ^2}{L(t)^2}+\frac{{\dot{A}}(t)^2}{4D}+\frac{{\underline{Q}}(t)}{2D}\right) dt. \end{aligned}$$

### Proof

By comparing (35) with (33) it follows that

40 $$\begin{aligned} \frac{1}{T}\int _0^T \left( \frac{D\pi ^2}{L(t)^2} -\frac{{\overline{Q}}(t)}{2D}\right) dt \le \mu _w \le \frac{1}{T}\int _0^T \left( \frac{D\pi ^2}{L(t)^2}+\frac{{\underline{Q}}(t)}{2D}\right) dt. \end{aligned}$$

The result then follows immediately by combining this with Lemma 1. $\square$

### Remark 2

If we were instead interested in the principal periodic eigenvalue $\mu _V$ of an operator $\frac{\partial }{\partial t}-D\nabla ^2+V(x,t)$ on $\Omega (t)$, where *V* was a continuous function on $\Omega (t)$ and periodic in *t* with the same period as the domain, then a similar result could be derived provided that ${\overline{Q}}(t)$ and ${\underline{Q}}(t)$ were adjusted appropriately to also include the terms from *V*.

### Example 3

Let $L(t)=L_0(1+\varepsilon \sin (\omega t))$ with $\omega >0$, $0<\varepsilon <1$, and consider the domain $\Omega (t)=(0,L(t))$ which has period $T=\frac{2\pi }{\omega }$. Let $\mu =\mu _u$ be the principal periodic eigenvalue of $\Omega (t)$. We shall apply Theorems 1, 2 and 4 to give some bounds on $\mu$. First we must consider

41 $$\begin{aligned} s(t):=\int \limits _0^t \frac{L_0^2}{L(\zeta )^2}d\zeta =\int \limits _0^t \frac{1}{(1+\varepsilon \sin (\omega \zeta ))^2}d\zeta . \end{aligned}$$

This integral (41) can be calculated exactly. For $-\frac{\pi }{\omega }< t<\frac{\pi }{\omega }$,

$$\begin{aligned} s(t)=&\frac{2}{\omega (1-\varepsilon ^2)^{3/2}}\left( \arctan \left( \frac{\tan (\frac{\omega t}{2})+\varepsilon }{\sqrt{1-\varepsilon ^2}}\right) - \arctan \left( \frac{\varepsilon }{\sqrt{1-\varepsilon ^2}}\right) \right) -\frac{2\varepsilon }{\omega (1-\varepsilon ^2)} \\&+\frac{2\varepsilon ^2\tan (\frac{\omega t}{2})+2\varepsilon }{\omega (1-\varepsilon ^2)\left( (\tan (\frac{\omega t}{2})+\varepsilon )^2+1-\varepsilon ^2\right) } . \end{aligned}$$

For $$t=\pm \frac{\pi }{\omega }$$,

$$\begin{aligned} s\left( \pm \frac{\pi }{\omega }\right) =\frac{2}{\omega (1-\varepsilon ^2)^{3/2}}\left( \pm \frac{\pi }{2} - \arctan \left( \frac{\varepsilon }{\sqrt{1-\varepsilon ^2}}\right) \right) -\frac{2\varepsilon }{\omega (1-\varepsilon ^2)}, \end{aligned}$$

and for $t> \frac{\pi }{\omega }$ we can use the fact that the integrand of *s*(*t*) is periodic. Note that at $t=\frac{2\pi }{\omega }$,

42 $$\begin{aligned} s\left( \frac{2\pi }{\omega }\right) =\frac{2\pi }{\omega }\frac{1}{ (1-\varepsilon ^2)^{3/2}}. \end{aligned}$$

By the periodicity, it follows that

43 $$\begin{aligned} s(t)=\int \limits _0^t \frac{L_0^2}{L(\zeta )^2}d\zeta =\frac{t}{(1-\varepsilon ^2)^{3/2}}+O(1) \qquad \text {as }t\rightarrow \infty . \end{aligned}$$

Therefore we conclude from Theorems 1 and 2 that

44 $$\begin{aligned} \frac{D\pi ^2}{L_0^2(1-\varepsilon ^2)^{3/2}} \le \mu \le \frac{D\pi ^2}{L_0^2(1-\varepsilon )^2}. \end{aligned}$$

This means that, regardless of the frequency $\omega$, the solution to the linear version of (1), (2), will tend to zero if $f'(0)<\frac{D\pi ^2}{L_0^2(1-\varepsilon ^2)^{3/2}}$, or tend to infinity if $f'(0)> \frac{D\pi ^2}{L_0^2(1-\varepsilon )^2}$.

To apply Theorem 4, we calculate the $\frac{2\pi }{\omega }$-periodic functions ${\overline{Q}}(t)$ and ${\underline{Q}}(t)$ as defined in (32):

$$\begin{aligned} {\overline{Q}}(t)= & {} {\left\{ \begin{array}{ll} 0 &{}\text {for }0\le t\le \frac{\pi }{\omega }\\ \displaystyle {-\frac{L_0^2\varepsilon \omega ^2}{2}\sin (\omega t)(1+\varepsilon \sin (\omega t))}&{}\text {for }\frac{\pi }{\omega }\le t\le \frac{2\pi }{\omega }\\ \end{array}\right. }\\ {\underline{Q}}(t)= & {} {\left\{ \begin{array}{ll}\displaystyle {\frac{L_0^2\varepsilon \omega ^2}{2}\sin (\omega t)(1+\varepsilon \sin (\omega t))}&{}\text {for } 0\le t\le \frac{\pi }{\omega }\\ 0&{}\text {for }\frac{\pi }{\omega }\le t\le \frac{2\pi }{\omega }\\ \end{array}\right. } \end{aligned}$$

and therefore

$$\begin{aligned} \int _0^{\frac{2\pi }{\omega }} \frac{{\overline{Q}}(\zeta )}{2D}d\zeta = \frac{L_0^2\varepsilon \omega }{2D}\left( 1-\frac{\varepsilon \pi }{4}\right) \qquad \text {and}\qquad \int _0^{\frac{2\pi }{\omega }} \frac{{\underline{Q}}(\zeta )}{2D}d\zeta = \frac{L_0^2\varepsilon \omega }{2D}\left( 1+\frac{\varepsilon \pi }{4}\right) . \end{aligned}$$

By Eq. (39) together with (43) we deduce that

45 $$\begin{aligned} \frac{D\pi ^2}{L_0^2(1-\varepsilon ^2)^{3/2}} -\frac{L_0^2\varepsilon \omega ^2}{4\pi D}\left( 1-\frac{\varepsilon \pi }{4}\right) \le \mu \le \frac{D\pi ^2}{L_0^2(1-\varepsilon ^2)^{3/2}}+ \frac{L_0^2\varepsilon \omega ^2}{4\pi D}\left( 1+\frac{\varepsilon \pi }{4}\right) , \end{aligned}$$

although we note that in this case the lower bound in (44) is better.

### Remark 3

Let $\mu (\omega )$ be the principal periodic eigenvalue of $\Omega (t)=(0,L(t))$ with *L*(*t*) as in Example 3 for some fixed $\varepsilon \in (0,1)$. The bounds (45) imply that

46 $$\begin{aligned} \mu (\omega )= \frac{D\pi ^2}{L_0^2(1-\varepsilon ^2)^{3/2}}+O(\omega ^2)=\frac{1}{T}\int \limits _0^T\frac{D\pi ^2}{L(t)^2}dt+O(\omega ^2) \quad \text { as }\omega \rightarrow 0. \end{aligned}$$

In fact, for any interval of the form $A(t)<x<A(t)+L(t)$ where $A(t)=A_1 \left( \frac{\omega t}{2\pi }\right)$ and $L(t)=L_1\left( \frac{\omega t}{2\pi }\right)$ for some smooth and 1-periodic functions $L_1>0$ and $A_1$, we can use (39) to deduce the limit as $\omega \rightarrow 0$. Indeed since ${\dot{A}}^2$, ${\underline{Q}}$ and ${\overline{Q}}$ (as defined in (32)) are all $O(\omega ^2)$ as $\omega \rightarrow 0$, we can conclude from (39) that

47 $$\begin{aligned} \mu (\omega ) =\int _0^1 \frac{D\pi ^2}{L_1(s)^2}ds + O(\omega ^2) \qquad \text {as }\omega \rightarrow 0. \end{aligned}$$

In the next section we shall see that the limit $\lim _{\omega \rightarrow 0} \mu (\omega )$ from (46) and (47) is an instance of a more general property, which is valid for $\frac{2\pi }{\omega }$-periodic domains $\Omega (t)$ in any dimension, as $\omega \rightarrow 0$.

## Dependence of $\mu$ on the Frequency $\omega$

### Converting to a 1-Periodic Problem

In this section we consider the principal periodic eigenvalue $\mu$ as a function of the frequency $\omega =\frac{2\pi }{T}$. We consider a 1-periodic domain ${\tilde{\Omega }}(s)$ and let $\mu =\mu (\omega )$ be the principal periodic eigenvalue associated with the domain

48 $$\begin{aligned} \Omega (t)={\tilde{\Omega }}\left( \frac{\omega t}{2\pi }\right) . \end{aligned}$$

Note that the map $h(\cdot ,t):\Omega (t)\rightarrow \Omega _0$ which we used in the change of variables can now be expressed as $h(\cdot ,t)={\tilde{h}}(\cdot ,\frac{\omega t}{2\pi })$, for a 1-periodic map ${\tilde{h}}(\cdot ,s): {\tilde{\Omega }}(s)\rightarrow \Omega _0$. If we change variables from *t* to $s=\frac{\omega t}{2\pi }$ in (10), (11), then the operator $\frac{\partial }{\partial t}- {\mathcal {L}}(\xi ,t)$ becomes an operator of the form $\frac{\omega }{2\pi }\frac{\partial }{\partial s}- {\mathcal {L}}_{\omega }(\xi ,s)$ where

49 $$\begin{aligned} {\mathcal {L}}_{\omega }(\xi ,s) = \sum _{i,j}{\tilde{a}}_{ij}(\xi ,s)\frac{\partial ^2 u}{\partial \xi _i\partial \xi _j} +\sum _j \left( \frac{\omega }{2\pi }{\tilde{b}}_j(\xi ,s) +{\tilde{c}}_j(\xi ,s)\right) \frac{\partial }{\partial \xi _j}, \end{aligned}$$

50 $$\begin{aligned} {\tilde{a}}_{ij}(\xi ,s)= \sum _{k}D\left( \frac{\partial {\tilde{h}}_i}{\partial x_k}\frac{\partial {\tilde{h}}_j}{\partial x_k}\right) , \qquad {\tilde{b}}_j(\xi ,s)= -\frac{\partial {\tilde{h}}_j}{\partial s}, \qquad {\tilde{c}}_j(\xi ,s)=D\nabla ^2 {\tilde{h}}_j. \end{aligned}$$

So the principal periodic eigenvalue of $\Omega (t)={\tilde{\Omega }}\left( \frac{\omega t}{2\pi }\right)$ is the same as the principal periodic eigenvalue $\mu (\omega )$ of the problem

51 $$\begin{aligned} \frac{\omega }{2\pi }\frac{\partial \phi }{\partial s}- {\mathcal {L}}_{\omega }(\xi ,s)\phi = \mu (\omega )\phi (\xi ,s) \qquad \xi \in \Omega _0, s\in [0,1] \end{aligned}$$

52 $$\begin{aligned} \phi (\xi ,s)=0 \qquad \xi \in \partial \Omega _0, s\in [0,1] \end{aligned}$$

53 $$\begin{aligned} \phi (\xi ,s)\equiv \phi (\xi ,s+1) \qquad \xi \in \Omega _0. \end{aligned}$$

For any $\omega >0$, the coefficients of ${\mathcal {L}}_{\omega }(\xi ,s)$ are 1-periodic in *s*. However, the term $\frac{\omega }{2\pi } {\tilde{b}}_j(\xi ,s) \frac{\partial }{\partial \xi _j}$ in (49) still depends on, and scales with, $\omega$. The source of this term is the coefficient involving $\frac{\partial h_j}{\partial t}=\frac{\omega }{2\pi }\frac{\partial {\tilde{h}}_j}{\partial s}$ which we get when we transform the time-dependent domain ${\tilde{\Omega }}\left( \frac{\omega t}{2\pi }\right)$ into the fixed reference domain $\Omega _0$.

In the paper [9], Liu et al. consider parabolic equations with periodic coefficients, and they investigate how the principal periodic eigenvalue varies with respect to the frequency. However, the coefficients in their equation are independent of the frequency $\omega$ except where it appears inside the periodic functions as $\omega t$. Therefore, after the change of time variables to give an operator with 1-periodic coefficients, the problems they consider have the form

54 $$\begin{aligned} \frac{\omega }{2\pi }\frac{\partial {\hat{\phi }}}{\partial s}- \mathcal {{\hat{L}}}(\xi ,s){\hat{\phi }} = {\hat{\lambda }}(\omega ){\hat{\phi }}(\xi ,s) \qquad \xi \in \Omega _0, s\in [0,1] \end{aligned}$$

55 $$\begin{aligned} {\hat{\phi }}(\xi ,s)=0 \qquad \xi \in \partial \Omega _0, s\in [0,1] \end{aligned}$$

56 $$\begin{aligned} {\hat{\phi }}(\xi ,s)\equiv {\hat{\phi }}(\xi ,s+1) \qquad \xi \in \Omega _0, \end{aligned}$$

where the coefficients of the operator $\mathcal {{\hat{L}}}(\xi ,s)$ are 1-periodic in *s* and do not depend on $\omega$. We shall use methods from [9] in the proofs of Theorem 5 and Theorem 7, but the methods have to be adapted, since the operator ${\mathcal {L}}_{\omega }(\xi ,s)$ depends on $\omega$ through the $\frac{\omega }{2\pi } {\tilde{b}}_j(\xi ,s) \frac{\partial }{\partial \xi _j}$ term.

### Asymptotic Behaviour of $\mu (\omega )$ as $\omega \rightarrow 0$

In this section, we consider the limit of $\mu (\omega )$ as $\omega \rightarrow 0$. The proof of Theorem 5 is essentially the same as that used in [9, Theorem 1.3(i)] to prove Eq. (8), however a slight generalisation is needed to allow for the $\omega$ dependence of the coefficients in $-{\mathcal {L}}_{\omega }(\xi ,s)$. For completeness, we give the proof here.

#### Theorem 5

Let $\Omega _0$ be a smooth bounded domain, and for each $s \in [0,1]$ and $\omega \ge 0$ let $-{\mathcal {L}}_{\omega }(\xi ,s)$ be as defined in Eq. (49). Assume the coefficients ${\tilde{a}}_{ij}$, ${\tilde{b}}_j$, ${\tilde{c}}_j$ belong to $C^{1+\alpha , 1+\alpha }(\overline{\Omega _0}\times [0,1])$ for some $\alpha >0$.

For $s \in [0,1]$ and $\omega \ge 0$, let $\lambda ^{0}(s,\omega )$ be the principal eigenvalue of the elliptic operator $-{\mathcal {L}}_{\omega }(\xi ,s)$ on $\Omega _0$, with zero Dirichlet conditions on $\partial \Omega _0$. For $\omega >0$, let $\mu (\omega )$ be the principal periodic eigenvalue of (51), (52), (53). Then

57 $$\begin{aligned} \lim _{\omega \rightarrow 0}\mu (\omega )= \int _0^1 \lambda ^{0}(s,0)ds. \end{aligned}$$

#### Proof

For each $s \in [0,1]$ and $\omega \ge 0$, let $\lambda ^{0}(s,\omega )$ and $\phi ^{0}(\xi ; s,\omega )$ be the principal eigenvalue and eigenfunction of the elliptic operator $-{\mathcal {L}}_{\omega }(\xi ,s)$ on $\Omega _0$, with zero Dirichlet conditions on $\partial \Omega _0$, and normalised to $\vert \vert \phi ^{0}(\cdot \; s,\omega ) \vert \vert _{L^2(\Omega _0)} =1$. As in [9], note that for any $\xi \in \overline{\Omega _0}$ and $\omega \ge 0$, both $\phi ^{0}(\xi ;s,\omega )$ and $\nabla \phi ^{0}(\xi ;s,\omega )$ are $C^1$ and 1-periodic in *s*. We also note here that they, and $\lambda ^{0}(s,\omega )$, depend continuously on $\omega$.

For $$\omega >0$$, define

58 $$\begin{aligned} {\overline{\phi }}_{\omega }(\xi ,s) = \phi ^{0}(\xi ; s,\omega ) \rho _{\omega }(s) \end{aligned}$$

where

59 $$\begin{aligned} \rho _{\omega }(s)=\exp \left( \frac{2\pi }{\omega }\left( s \int _0^1 \lambda ^{0}(\tau ,\omega ) d\tau - \int _0^s \lambda ^{0}(\tau ,\omega ) d\tau \right) \right) . \end{aligned}$$

Note that $\rho _{\omega }>0$, $\rho _{\omega }$ is periodic with period 1, and satisfies

60 $$\begin{aligned} \frac{\omega }{2\pi } \frac{d \rho _{\omega }}{ds}=\left( \int _0^1 \lambda ^{0}(\tau ,\omega ) d\tau - \lambda ^{0}(s,\omega ) \right) \rho _{\omega }(s). \end{aligned}$$

We shall show that given $\varepsilon >0$, $\omega _{\varepsilon }>0$ can be chosen small enough such that

61 $$\begin{aligned} \left( \int _0^1 \lambda ^{0}(\tau ,\omega ) d\tau -\varepsilon \right) {\overline{\phi }}_{\omega } \le \frac{\omega }{2\pi }\frac{\partial {\overline{\phi }}_{\omega }}{\partial s}- {\mathcal {L}}_{\omega }(\xi ,s){\overline{\phi }}_{\omega } \le \left( \int _0^1 \lambda ^{0}(\tau ,\omega ) d\tau +\varepsilon \right) {\overline{\phi }}_{\omega }\nonumber \\ \end{aligned}$$

for all $0<\omega \le \omega _{\varepsilon }$. Then since ${\overline{\phi }}_{\omega }$ is positive, 1-periodic in *s*, and satisfies the Dirichlet boundary conditions on $\partial \Omega _0$, it follows from [11, Proposition 2.1] that

62 $$\begin{aligned} \int _0^1 \lambda ^{0}(\tau ,\omega ) d\tau -\varepsilon \le \mu (\omega ) \le \int _0^1 \lambda ^{0}(\tau ,\omega ) d\tau +\varepsilon \qquad \text {for all }0<\omega \le \omega _{\varepsilon }, \end{aligned}$$

and so we reach the conclusion

63 $$\begin{aligned} \lim _{\omega \rightarrow 0} \left( \mu (\omega ) - \int _0^1 \lambda ^{0}(s,\omega ) ds \right) =0. \end{aligned}$$

Finally, since $\lambda ^{0}(s,\omega )$ depends continuously on $\omega$, (63) implies (57).

It remains to show that $\omega _{\varepsilon }$ can be chosen such that (61) holds for all $0<\omega \le \omega _{\varepsilon }$. Using (58), (60), and the fact that $\phi ^{0}(\xi ; s,\omega )$ is an eigenfunction of $-{\mathcal {L}}_{\omega }(\xi ,s)$ with eigenvalue $\lambda ^{0}(s,\omega )$, we calculate

64 $$\begin{aligned} \frac{\omega }{2\pi }\frac{\partial {\overline{\phi }}_{\omega }}{\partial s}- {\mathcal {L}}_{\omega }(\xi ,s){\overline{\phi }}_{\omega }=&\frac{\omega }{2\pi }\frac{\partial \phi ^{0}(\xi ; s,\omega )}{\partial s} \rho _{\omega } \nonumber \\&+ \frac{\omega }{2\pi } \frac{d \rho _{\omega }}{ds}\phi ^{0}(\xi ; s,\omega ) + \lambda ^{0}(s,\omega )\phi ^{0}(\xi ; s,\omega )\rho _{\omega } \end{aligned}$$

65 $$\begin{aligned} =&\left( \frac{\omega }{2\pi }\frac{\partial \phi ^{0}(\xi ; s,\omega )}{\partial s} + \int _0^1 \lambda ^{0}(\tau ,\omega ) d\tau \ \phi ^{0}(\xi ; s,\omega ) \right) \rho _{\omega }. \end{aligned}$$

Therefore (61) will hold provided that we can choose $\omega _{\varepsilon }>0$ such that

66 $$\begin{aligned} \frac{\omega }{2\pi }\left| \frac{\partial \phi ^{0}(\xi ; s,\omega )}{\partial s} \right| \le \varepsilon \phi ^{0}(\xi ; s,\omega ) \qquad \text {for all } \xi \in \Omega _0,\ s\in [0,1],\ 0<\omega \le \omega _{\varepsilon }. \end{aligned}$$

Since $\phi ^{0}(\xi ; s,\omega )$ is positive in $\Omega _0$, we know that $\frac{\frac{\partial \phi ^{0}}{\partial s}(\xi ; s,\omega )}{\phi ^{0}(\xi ; s,\omega )}$ is finite for each $\xi$ in $\Omega _0$. For any point $\xi _0\in \partial \Omega _0$, with outward normal $\nu$, we may consider a sequence $\xi \in \Omega _0$, $\xi \rightarrow \xi _0$ with $\frac{\xi -\xi _0}{\vert \xi -\xi _0\vert }\cdot \nu \nrightarrow 0$. Hopf’s Lemma implies that $\nabla \phi ^{0}(\xi _0; s,\omega )\cdot \nu \ne 0$, and then by l’Hôpital’s rule, we have

67 $$\begin{aligned} \lim _{\xi \rightarrow \xi _0} \frac{\frac{\partial \phi ^{0}}{\partial s}(\xi ; s,\omega )}{\phi ^{0}(\xi ; s,\omega )} =\frac{\nabla \frac{\partial \phi ^{0}}{\partial s}(\xi _0; s,\omega )\cdot \nu }{\nabla \phi ^{0}(\xi _0; s,\omega )\cdot \nu }= O(1). \end{aligned}$$

By continuity, and by the compactness of $\overline{\Omega _0}\times [0,1]\times [0,1]$, we find that $\frac{\frac{\partial \phi ^{0}}{\partial s}(\xi ; s,\omega )}{\phi ^{0}(\xi ; s,\omega )}$ is bounded uniformly with respect to $(\xi _0,s,\omega )\in \Omega _0\times [0,1]\times [0,1]$. Therefore $\omega _{\varepsilon }>0$ can be chosen to satisfy (66). $\square$

#### Corollary 1

Let ${\tilde{\Omega }}(s)$ be a smooth bounded domain which varies smoothly and 1-periodically with *s*, and let $\mu (\omega )$ be the principal periodic eigenvalue of $\Omega (t)={\tilde{\Omega }}\left( \frac{\omega t}{2\pi }\right)$. For each $0\le s\le 1$, let $\lambda ({\tilde{\Omega }}(s))$ be the principal Dirichlet eigenvalue of $-\nabla ^2$ on ${\tilde{\Omega }}(s)$. Then

68 $$\begin{aligned} \lim _{\omega \rightarrow 0}\mu (\omega ) =\int _0^1 D\lambda ({\tilde{\Omega }}(s))ds. \end{aligned}$$

#### Proof

For each $0\le s\le 1$, the change of variables ${\tilde{h}}$ from ${\tilde{\Omega }}(s)$ to $\Omega _0$ transforms the operator $D\nabla ^2$ on ${\tilde{\Omega }}(s)$ to $\sum _{i,j,k} D\left( \frac{\partial {\tilde{h}}_i}{\partial x_k}\frac{\partial {\tilde{h}}_j}{\partial x_k}\right) \frac{\partial ^2 }{\partial \xi _i\partial \xi _j} +\sum _jD\nabla ^2 {\tilde{h}}_j\frac{\partial }{\partial \xi _j}$ on $\Omega _0$. By Eqs. (49) and (50), this is precisely ${\mathcal {L}}_{0}(\xi ,s)$ (i.e. ${\mathcal {L}}_{\omega }(\xi ,s)$ with $\omega =0$). So we have

69 $$\begin{aligned} D\lambda ({\tilde{\Omega }}(s))=\lambda ^{0}(s,0) \end{aligned}$$

and then (68) is equivalent to (57). $\square$

#### Remark 4

Recall that $\int _0^1 D\lambda ({\tilde{\Omega }}(s))ds$ is also a lower bound for $\mu (\omega )$ for every $\omega >0$ (see Theorem 1).

#### Remark 5

A result corresponding to Corollary 1 would also hold for the principal periodic eigenvalue $\mu _V$ of an operator $\frac{\partial }{\partial t}-D\nabla ^2+V(x,t)$ on $\Omega (t)$, where *V* was a continuous function on $\Omega (t)$ and periodic in *t* with the same period as the domain. Writing $V(x,t)={\tilde{V}}\left( x,\frac{\omega t}{2\pi }\right)$, the same proof would show then that

$$\begin{aligned} \lim _{\omega \rightarrow 0}\mu _V(\omega ) =\int _0^1 \lambda _{D,{\tilde{V}}}({\tilde{\Omega }}(s))ds \end{aligned}$$

where for each *s*, $\lambda _{D,{\tilde{V}}}({\tilde{\Omega }}(s))$ was the principal Dirichlet eigenvalue of the elliptic operator $-D\nabla ^2+{\tilde{V}}(x,s)$ on ${\tilde{\Omega }}(s)$.

### Asymptotic Behaviour of $\mu (\omega )$ as $\omega \rightarrow \infty$

For one-dimensional time-periodic domains $\Omega (t)=(A(t),A(t)+L(t))$, we shall give conditions under which $\mu (\omega )$ does and does not remain bounded as $\omega \rightarrow \infty$. We also discuss how to extend such results to certain periodic domains in higher dimensions.

#### Theorem 6

Let $l(\cdot )$ and $a(\cdot )$ be 1-periodic functions, belonging to $C^{2+\alpha }([0,1])$ for some $\alpha >0$, and with $\min _{[0,1]} l=1$, $\max _{[0,1]} \vert a\vert =1$. For some $L_0>0$, $A_0\ge 0$, $\omega >0$, let

70 $$\begin{aligned} L(t)=L_0 l\left( \frac{\omega t}{2\pi }\right) , \qquad A(t)=A_0 a\left( \frac{\omega t}{2\pi }\right) , \end{aligned}$$

and let $\mu (\omega )=\mu _u(\omega )$ be the principal periodic eigenvalue associated with $\Omega (t)=(A(t),A(t)+L(t))$. Then $\mu (\omega )=O(\omega ^2)$ as $\omega \rightarrow \infty$, and if $a(\cdot )$ is constant, then $\mu (\omega )=O(1)$ as $\omega \rightarrow \infty$. Moreover, if $a(\cdot )$ is non-constant, there exist constants $C_1$, $C_2$ depending only on the functions *l* and *a* such that

1. If $\frac{A_0}{L_0}<C_1$ then $\mu (\omega )=O(1)$ as $\omega \rightarrow \infty$.
2. If $\frac{A_0}{L_0}>C_2$ then $\mu (\omega )=\underline{{\overline{O}}}(\omega ^2)$ as $\omega \rightarrow \infty$.

As before, the notation $\mu (\omega )=\underline{{\overline{O}}}(\omega ^2)$ is used to mean that $\mu (\omega )$ is ‘exactly of order’ $\omega ^2$ in the sense that $\mu (\omega )=O(\omega ^2)$ and $\omega ^2=O(\mu (\omega ))$ as $\omega \rightarrow \infty$.

#### Proof

If $\max _{s\in [0,1]} (A_0 a(s))< \min _{s\in [0,1]}(A_0 a(s)+L_0 l(s))$ then by Theorems 1 and 2, we have lower and upper bounds on $\mu (\omega )$ which are independent of $\omega >0$:

71 $$\begin{aligned} \frac{D\pi ^2}{L_0^2}\int _0^1 \frac{1}{l(s)^2}ds \le \mu (\omega ) \le \frac{D\pi ^2}{(\min _{[0,1]}(A_0 a+L_0 l) - \max _{[0,1]} (A_0 a))^2}. \end{aligned}$$

So, $\mu (\omega )=O(1)$ as $\omega \rightarrow \infty$ as long as $\max _{[0,1]} (A_0 a)< \min _{[0,1]}(A_0 a+L_0 l)$. If $a(\cdot )$ is constant then this will be satisfied because, by assumption, $\min _{[0,1]} (L_0 l)>0$. If $a(\cdot )$ is non-constant, then a sufficient condition is that

72 $$\begin{aligned} \frac{A_0}{L_0}<\frac{\min l}{\max a -\min a}. \end{aligned}$$

Next, in order to prove the other claimed properties, we shall consider the bounds (39) that were proved in Theorem 4. Define non-negative constants $c_1$, $c_2$, $c_3$, $c_4$, $c_5$, $c_6$ in terms of the functions *l* and *a* as follows:

$$\begin{aligned} \begin{array}{llll} &{}\displaystyle c_1=\int _0^1 \frac{1}{l(s)^2}ds, &{}\qquad \displaystyle c_2=\int _0^1 a'(s)^2 ds, &{}\qquad \displaystyle c_3=\int _0^1 l(s)[a''(s)]^{+} ds, \\ &{}\displaystyle c_4=\int _0^1 l(s)[l''(s)]^{+} ds, &{}\displaystyle \qquad c_5=\int _0^1 l(s)[a''(s)]^{-} ds, &{}\qquad \displaystyle c_6=\int _0^1 l(s)[l''(s)]^{-} ds. \end{array} \end{aligned}$$

Then note that

73 $$\begin{aligned} \frac{1}{T}\int _0^T \frac{D\pi ^2}{L(t)^2}dt=\frac{D\pi ^2}{L_0^2} c_1, \end{aligned}$$

74 $$\begin{aligned} \frac{1}{T}\int _0^T \frac{{\dot{A}}(t)^2}{4D}dt=\left( \frac{\omega }{2\pi }\right) ^2 \frac{A_0^2}{4D} c_2, \end{aligned}$$

75 $$\begin{aligned} 0\le \frac{1}{T}\int _0^T\frac{{\overline{Q}}(t)}{2D} dt \le \left( \frac{\omega }{2\pi }\right) ^2 \left( \frac{A_0L_0}{2D}c_3 +\frac{L_0^2}{4D}c_4 \right) , \end{aligned}$$

76 $$\begin{aligned} 0\le \frac{1}{T}\int _0^T\frac{{\underline{Q}}(t)}{2D}dt \le \left( \frac{\omega }{2\pi }\right) ^2 \left( \frac{A_0L_0}{2D}c_5 + \frac{L_0^2}{4D}c_6\right) . \end{aligned}$$

Therefore Theorem 4 implies that

77 $$\begin{aligned} \frac{D\pi ^2}{L_0^2}c_1 +&\left( \frac{\omega }{2\pi }\right) ^2\left( \frac{A_0^2}{4D}c_2 -\frac{A_0L_0}{2D}c_3 -\frac{L_0^2}{4D}c_4\right) \nonumber \\&\le \mu (\omega ) \le \frac{D\pi ^2}{L_0^2}c_1 +\left( \frac{\omega }{2\pi }\right) ^2\left( \frac{A_0^2}{4D}c_2 +\frac{A_0L_0}{2D}c_5 +\frac{L_0^2}{4D}c_6\right) , \end{aligned}$$

which proves that $\mu (\omega )=O(\omega ^2)$ as $\omega \rightarrow \infty$, for any $A_0$, $L_0$. Moreover, if the inequality $\frac{A_0^2}{4}c_2 -\frac{A_0L_0}{2}c_3 -\frac{L_0^2}{4}c_4 >0$ holds then $\mu (\omega )=\underline{{\overline{O}}}(\omega ^2)$ as $\omega \rightarrow \infty$. If $a(\cdot )$ is non-constant then $c_2\ne 0$ and so this inequality will hold for $\frac{A_0}{L_0}$ large enough (depending on $c_2$, $c_3$, $c_4$).

$$\square $$

In the following example we give these estimates explicitly.

#### Example 4

Let $L_0>0$ be constant and $A(t)=A_0\sin (\omega t)$ for some $\omega >0$, $A_0>0$. Consider the $\frac{2\pi }{\omega }$-periodic domain $\Omega (t)=(A(t),A(t)+L_0)$ and let $\mu (\omega )=\mu _u(\omega )$ be the principal periodic eigenvalue on $\Omega (t)$. By Theorems 1 and 2 we conclude that

78 $$\begin{aligned} \frac{D\pi ^2}{L_0^2} \le \mu (\omega ) \qquad \text {for every } \omega >0, \end{aligned}$$

79 $$\begin{aligned} \text {and if }2A_0<L_0, \qquad \mu (\omega )\le \frac{D\pi ^2}{(L_0-2A_0)^2} \qquad \text {for every } \omega >0. \end{aligned}$$

To apply the bounds from Theorem 4, calculate

80 $$\begin{aligned} \int \limits _0^t \frac{{\dot{A}}(\zeta )^2}{4D} d\zeta =\frac{A_0^2\omega ^2}{4D} \left( \frac{t}{2}+\frac{\sin (2\omega t)}{4\omega }\right) . \end{aligned}$$

Also calculate the $\frac{2\pi }{\omega }$-periodic functions ${\overline{Q}}(t)$ and ${\underline{Q}}(t)$ as defined in (32):

$$\begin{aligned} {\overline{Q}}(t)= & {} {\left\{ \begin{array}{ll} 0 &{}\text {for }0\le t\le \frac{\pi }{\omega }\\ \displaystyle {-A_0L_0\omega ^2\sin (\omega t)}&{}\text {for }\frac{\pi }{\omega }\le t\le \frac{2\pi }{\omega }, \end{array}\right. } \\ {\underline{Q}}(t)= & {} {\left\{ \begin{array}{ll}\displaystyle {A_0L_0\omega ^2\sin (\omega t)}&{}\text {for } 0\le t\le \frac{\pi }{\omega }\\ 0&{}\text {for }\frac{\pi }{\omega }\le t\le \frac{2\pi }{\omega },\\ \end{array}\right. } \end{aligned}$$

and so

$$\begin{aligned} \int _0^{\frac{2\pi }{\omega }} \frac{{\overline{Q}}(\zeta )}{2D}d\zeta = \int _0^{\frac{2\pi }{\omega }} \frac{{\underline{Q}}(\zeta )}{2D}d\zeta = \frac{A_0 L_0\omega }{D}. \end{aligned}$$

By Theorem 4 we deduce that

81 $$\begin{aligned} \frac{D\pi ^2}{L_0^2}+\frac{A_0^2\omega ^2}{8D}-\frac{A_0 L_0 \omega ^2}{2\pi D} \le \mu (\omega ) \le \frac{D\pi ^2}{L_0^2} +\frac{A_0^2\omega ^2}{8D}+\frac{A_0 L_0 \omega ^2}{2\pi D}. \end{aligned}$$

In agreement with Corollary 1 and Theorem 6, the bounds (78), (79) and (81) show that:

$$\begin{aligned} \mu =\frac{D\pi ^2}{L_0^2}+O(\omega ^2) \text { as }\omega \rightarrow 0. \\ \mu (\omega )=O(\omega ^2)\text { as }\omega \rightarrow \infty . \\ \text {If } \quad \frac{A_0}{L_0} < \frac{1}{2} \quad \text { then} \quad \mu (\omega )=O(1)\text { as }\omega \rightarrow \infty . \\ \text {If } \quad \frac{A_0}{L_0}> \frac{4}{\pi }\quad \text { then} \quad \mu (\omega )=\underline{{\overline{O}}}(\omega ^2)\text { as }\omega \rightarrow \infty . \end{aligned}$$

It would be interesting to investigate the $\omega \rightarrow \infty$ limit in the intermediate parameter range $\frac{L_0}{2}\le A_0 \le \frac{4L_0}{\pi }$.

Ideas from the proof of Theorem 6 can also be applied to multidimensional periodic domains $\Omega (t)={\tilde{\Omega }}\left( \frac{\omega t}{2\pi }\right)$ in ${\mathbb {R}}^N$. If there is some fixed domain $\Omega _1$ such that $\Omega _1 \subset {\tilde{\Omega }}(s)$ for all *s*, then it again follows easily from Theorems 1 and 2 that $\mu (\omega )=O(1)$ as $\omega \rightarrow \infty$. Consider next the case where ${\tilde{\Omega }}(s)$ is a box-like periodic domain of the form

$$\begin{aligned} A_{0,j}a_j(s)<x_j<A_{0,j}a_j(s)+L_{0,j}l_j(s) \qquad \quad j=1,\ldots ,N \end{aligned}$$

with $l_j(\cdot )$ and $a_j(\cdot )$ all 1-periodic functions, and with $\min _{[0,1]} l_j=1$, $\max _{[0,1]} \vert a_j\vert =1$, $L_{0,j}>0$, $A_{0,j}\ge 0$. Then a similar approach to that used above will give bounds resembling (77) but with all the terms summed over $j=1,\ldots ,N$. Consequently, we again find that $\mu (\omega )=O(\omega ^2)$ as $\omega \rightarrow \infty$, and that $\mu (\omega )=\underline{{\overline{O}}}(\omega ^2)$ as $\omega \rightarrow \infty$ if $\sum _{j=1}^N\left( \frac{A_{0,j}^2}{4}c_{2,j} -\frac{A_{0,j}L_{0,j}}{2}c_{3,j} -\frac{L_{0,j}^2}{4}c_{4,j}\right) >0$ where

$$\begin{aligned} c_{2,j}=\int _0^1 a_j'(s)^2 ds, \qquad c_{3,j}=\int _0^1 l_j(s)[a_j''(s)]^{+} ds, \qquad c_{4,j}=\int _0^1 l_j(s)[l_j''(s)]^{+} ds. \end{aligned}$$

If a more general periodic domain in ${\mathbb {R}}^N$ both encloses and is enclosed by such box-like periodic domains then it is possible to use this to prove conditions under which its own principal periodic eigenvalue $\mu (\omega )$ will also be $\underline{{\overline{O}}}(\omega ^2)$ as $\omega \rightarrow \infty$.

### Monotonicity of $\mu (\omega )$ with Respect to $\omega >0$

In this section, we prove first that the principal periodic eigenvalue associated with a *T*-periodic domain $\Omega (t)\subset {\mathbb {R}}^N$ is the same as the eigenvalue associated with the domain $\Omega (-t)$. We use this together with Lemma 2.1 and Theorem 1.1 from [9] to show that, for $\omega >0$, the principal periodic eigenvalue $\mu (\omega )$ associated with the domain ${\tilde{\Omega }}\left( \frac{\omega t}{2\pi }\right)$ is monotonic non-decreasing with respect to $\omega$.

#### Lemma 2

Let $\Omega (t)$ be a *T*-periodic domain. Let $\mu _+$ be the principal periodic eigenvalue associated with $\Omega (t)$, and let $\mu _-$ be the principal periodic eigenvalue associated with $\Omega _-(t):=\Omega (-t)$. Then $\mu _+= \mu _-$.

#### Proof

The eigenvalues $\mu _+$, $\mu _-$ are principal periodic eigenvalues of problems of the form (15), (16), (17), (18) on $\Omega _0$, with operators ${\mathcal {L}}_+$ and ${\mathcal {L}}_-$ coming from the changes of variables. In terms of the original co-ordinates, this means that there exist positive functions $\psi _+(x,t)$ on $\Omega (t)$ and $\psi _-(x,t)$ on $\Omega _-(t)$, which are *T*-periodic in *t*, and which satisfy

82 $$\begin{aligned} \frac{\partial \psi _+}{\partial t} = D \nabla ^2 \psi _+ + \mu _+\psi _+ \qquad \text {for }x\in \Omega (t) \end{aligned}$$

83 $$\begin{aligned} \psi _+(x,t)=0 \qquad \text {for }x\in \partial \Omega (t) \end{aligned}$$

84 $$\begin{aligned} \frac{\partial \psi _-}{\partial t} = D \nabla ^2 \psi _- + \mu _-\psi _- \qquad \text {for }x\in \Omega _-(t) \end{aligned}$$

85 $$\begin{aligned} \psi _-(x,t)=0 \qquad \text {for }x\in \partial \Omega _-(t). \end{aligned}$$

Let ${\overline{\psi }}(x,t)=\psi _-(x,-t)$ for $x\in \Omega _-(-t)=\Omega (t)$. Then ${\overline{\psi }}(x,t)$ is positive on $\Omega (t)$ and *T*-periodic in *t*, and satisfies

86 $$\begin{aligned} -\frac{\partial {\overline{\psi }}}{\partial t} = D \nabla ^2 {\overline{\psi }} + \mu _-{\overline{\psi }} \qquad \text {for }x\in \Omega (t) \end{aligned}$$

87 $$\begin{aligned} {\overline{\psi }}(x,t)=0 \qquad \text {for }x\in \partial \Omega (t). \end{aligned}$$

If $I(t)= \int _{\Omega (t)} \psi _+(x,t){\overline{\psi }}(x,t) dx$ then, using the zero Dirichlet boundary conditions on $\partial \Omega (t)$, we have

88 $$\begin{aligned} \frac{dI}{dt} =\int _{\Omega (t)} \left( \frac{\partial \psi _+}{\partial t}{\overline{\psi }} + \frac{\partial {\overline{\psi }}}{\partial t}\psi _+ \right) dx. \end{aligned}$$

Using Eqs. (82) and (86), and integrating by parts, this becomes

89 $$\begin{aligned} \frac{dI}{dt} = ( \mu _+ - \mu _-)I(t). \end{aligned}$$

Therefore $I(t)= I(0)e^{( \mu _+ - \mu _-)t}$. Since *I*(*t*) is periodic it must be that $\mu _+ - \mu _- =0$. $\square$

#### Remark 6

The proof of Lemma 2 not only shows that $\mu _+ = \mu _-$ but also that $I(t) \equiv I(0)$. That is, the integral $\int _{\Omega (t)} \psi _+(x,t)\psi _-(x,-t) dx$ is independent of *t*.

By rescaling time to $s = \frac{\omega t}{2\pi }$, we can now extend some ideas of Liu et al. in [9] to prove a monotonicity result with respect to the frequency $\omega$.

#### Theorem 7

Let ${\tilde{\Omega }}(s)$ be a 1-periodic domain, and $\omega >0$. Let $\mu (\omega )$ be the principal periodic eigenvalue associated with $\Omega (t)={\tilde{\Omega }}\left( \frac{\omega t}{2\pi }\right)$. Then $\mu (\omega )$ is monotonic non-decreasing: $\frac{d \mu (\omega )}{d\omega } \ge 0$. Moreover $\frac{d \mu (\omega )}{d\omega }=0$ if and only if the domain is independent of time.

#### Proof

Changing variables to $s = \frac{\omega t}{2\pi }$, the eigenfunctions $\psi _+(x,t)$ and ${\overline{\psi }}(x,t)$ from Lemma 2 now become functions $\phi _{\omega }(x,s)$ and ${\overline{\phi }}_{\omega }(x,s)$ which are positive on $x\in {\tilde{\Omega }}(s)$ and are 1-periodic in *s*. By Lemma 2 they satisfy

90 $$\begin{aligned} \frac{\omega }{2\pi } \frac{\partial \phi _{\omega }}{\partial s} = D \nabla ^2 \phi _{\omega } + \mu (\omega )\phi _{\omega } \qquad \text {for }x\in {\tilde{\Omega }}(s) \end{aligned}$$

91 $$\begin{aligned} -\frac{\omega }{2\pi } \frac{\partial {\overline{\phi }}_{\omega }}{\partial s} = D \nabla ^2 {\overline{\phi }}_{\omega } + \mu (\omega ){\overline{\phi }}_{\omega } \qquad \text {for }x\in {\tilde{\Omega }}(s) \end{aligned}$$

92 $$\begin{aligned} \phi _{\omega }(x,s)={\overline{\phi }}_{\omega }(x,s)=0 \qquad \text {for }x\in \partial {\tilde{\Omega }}(s). \end{aligned}$$

Without loss of generality we may normalise them so that

93 $$\begin{aligned} \int _0^1\int _{{\tilde{\Omega }}(s)} \phi _{\omega }(x,s)^2 dxds = \int _0^1\int _{{\tilde{\Omega }}(s)} \phi _{\omega }(x,s){\overline{\phi }}_{\omega }(x,s)dxds =1. \end{aligned}$$

Now, we follow the same steps as in [9, Theorem 1.1]. Namely we take Eq. (90) and differentiate it with respect to $\omega$. Writing $\phi _{\omega }'$ for $\frac{ \partial \phi _{\omega }}{\partial \omega }$ and $\mu '(\omega )$ for $\frac{d \mu (\omega )}{d \omega }$, this becomes

94 $$\begin{aligned} \frac{1}{2\pi } \frac{\partial \phi _{\omega }}{\partial s} + \frac{\omega }{2\pi } \frac{\partial \phi _{\omega }'}{\partial s} = D \nabla ^2 \phi _{\omega }' + \mu (\omega )\phi _{\omega }' + \mu (\omega )\phi _{\omega }\qquad \text {for }x\in {\tilde{\Omega }}(s). \end{aligned}$$

Multiply this by ${\overline{\phi }}_{\omega }$ and integrate over $x \in {\tilde{\Omega }}(s)$ and $s \in [0,1]$. Using the boundary conditions (92), the Eq. (91), and the normalisation, this leads to

95 $$\begin{aligned} \frac{1}{2\pi } \int _0^1 \int _{{\tilde{\Omega }}(s)} \frac{\partial \phi _{\omega }}{\partial s} {\overline{\phi }}_{\omega } \ dx ds = \mu '(\omega ). \end{aligned}$$

Next, we define a functional

96 $$\begin{aligned} J_{\omega }(\zeta ) = \int _0^1 \int _{{\tilde{\Omega }}(s)} \phi _{\omega }(x,s){\overline{\phi }}_{\omega }(x,s) \left( \frac{ \frac{\omega }{2\pi } \frac{\partial \zeta }{\partial s}- D \nabla ^2 \zeta }{\zeta (x,s)}\right) dxds \end{aligned}$$

for functions $\zeta (x,s)$ which are $C^2$ in $x\in {\tilde{\Omega }}(s)$ and $C^1$ on $\overline{{\tilde{\Omega }}(s)}$, and which are $C^1$ and 1-periodic in $s\in [0,1]$, and which are positive for $x\in {\tilde{\Omega }}(s)$, with $\zeta (x,s) =0$ and $\nabla \zeta \cdot \nu \ne 0$ for $x\in \partial {\tilde{\Omega }}(s)$ (see [9]). Then it is straightforward to check using (90) and (91) that Eq. (95) can be written as

97 $$\begin{aligned} \mu '(\omega ) = \frac{1}{2\pi } \int _0^1 \int _{{\tilde{\Omega }}(s)} \frac{\partial \phi _{\omega }}{\partial s} {\overline{\phi }}_{\omega } dx ds = \frac{1}{2\omega }\left( J_{\omega }(\phi _{\omega }) -J_{\omega }({\overline{\phi }}_{\omega }) \right) . \end{aligned}$$

To prove the required result we must therefore show that $J_{\omega }(\phi _{\omega }) -J_{\omega }({\overline{\phi }}_{\omega }) \ge 0$. But this can be proved exactly as in the proof of Lemma 2.1 from [9]. Indeed, although our definition of $J_{\omega }$ now includes a time-dependent domain ${\tilde{\Omega }}(s)$, one can check that each step of the proof of [9, Lemma 2.1, case 2 (b=1)] (i.e. the case of Dirichlet boundary conditions) goes through exactly as in [9]. This shows that, for all functions $\zeta$ of the class defined above,

98 $$\begin{aligned} J_{\omega }(\phi _{\omega }) -J_{\omega }(\zeta ) = \int _0^1 \int _{{\tilde{\Omega }}(s)} D \phi _{\omega }(x,s){\overline{\phi }}_{\omega }(x,s) \left| \nabla \left( \log \frac{\zeta }{\phi _{\omega }}\right) \right| ^2 dxds, \end{aligned}$$

and the right hand side is clearly non-negative. In particular, $J_{\omega }(\phi _{\omega }) -J_{\omega }({\overline{\phi }}_{\omega }) \ge 0$. This proves that $\mu '(\omega )\ge 0$. Moreover, $\mu '(\omega )=0$ if and only if $J_{\omega }(\phi _{\omega }) -J_{\omega }({\overline{\phi }}_{\omega }) = 0$, and by Eq. (98) this holds if and only if $\frac{{\overline{\phi }}_{\omega }}{\phi _{\omega }}$ is a function just of *s*. Substituting ${\overline{\phi }}_{\omega }(x,s)=\beta (s)\phi _{\omega }(x,s)$ into (91) and using (90) gives

99 $$\begin{aligned} \beta '(s)\phi _{\omega }+2\beta (s)\frac{\partial \phi _{\omega }}{\partial s}=0. \end{aligned}$$

Since $\phi _{\omega }=0$ on $\partial {\tilde{\Omega }}(s)$ and $\beta (s)>0$, this implies that also the (possibly one-sided) derivative $\frac{\partial \phi _{\omega }}{\partial s}$ is zero at each point *x* on $\partial {\tilde{\Omega }}(s)$. As $\phi _{\omega }>0$ on the interior and is zero with non-zero normal derivative on the boundary, we deduce that in fact the boundary $\partial {\tilde{\Omega }}(s)$ must remain the same for all times *s*: the domain is time-independent. Conversely, for all such time-independent domains, we have $\beta =1$ and $\mu$ does not depend on $\omega$. Thus $\mu '(\omega )=0$ if and only if the domain is independent of time. $\square$

#### Remark 7

It is possible to use the same proof to extend Lemma 2 to operators of the form $\frac{\partial }{\partial t}-D\nabla ^2+V(t)$ on $\Omega (t)$, where *V*(*t*) (independent of *x*) is a continuous and periodic function with the same period as the domain, and satisfies the extra condition that $V(t)\equiv V(-t)$. For operators of this form, the monotonicity result of Theorem 7 then also follows as above. However, the monotonicity results may not carry over to more general forms of the operator.

## Nonlinear Equation on a Periodic Domain

In this section, we consider the nonlinear periodic parabolic problem (10), (11) where *f* is assumed to satisfy the conditions (3). As above, let $\mu$ and $\phi (\xi ,t)$ be the principal periodic eigenvalue and eigenfunction satisfying (15), (16), (17), (18), and normalised so that $\vert \vert \phi \vert \vert _{\infty }=1$. Now the solution to the linear equation is a supersolution to the nonlinear problem, so if $f'(0)<\mu$ then $u\rightarrow 0$ as $t\rightarrow \infty$.

From now on, assume $f'(0)>\mu$. Fix any $\alpha \in (0, f'(0)-\mu )$. Then since $f(u)=f'(0)u+o(u)$ as $u\rightarrow 0$ there exists $\varepsilon >0$ (depending on $\alpha$) such that for all $0\le u\le \varepsilon$,

100 $$\begin{aligned} (\alpha - f'(0) +\mu )u +(f'(0)u-f(u))\le 0. \end{aligned}$$

Now, for any $\delta$ such that $0<\delta \le \varepsilon e^{-\alpha T}$, the function ${\hat{u}}(\xi ,t)=\delta \phi (\xi ,t)e^{\alpha t}$ is a subsolution for *u* for $0\le t\le T$:

101 $$\begin{aligned} \frac{\partial {\hat{u}}}{\partial t} - {\mathcal {L}}{\hat{u}} - f({\hat{u}})&= \alpha {\hat{u}} + \mu {\hat{u}}- f({\hat{u}}) \nonumber \\&= (\alpha - f'(0) +\mu ){\hat{u}} +(f'(0){\hat{u}}-f({\hat{u}})) \ \le 0 \end{aligned}$$

since ${\hat{u}}(\xi ,t)\le \varepsilon$ for $0\le t\le T$. The function ${\hat{u}}$ also satisfies ${\hat{u}}(\xi ,t)=0$ on $\partial \Omega _0$, and ${\hat{u}}(\xi ,0)\le {\hat{u}}(\xi ,T)$, and so it is a subsolution to the periodic problem (10), (11) in the sense of Hess [5, chapter III Definition 21.1]. Moreover the constant *K* is a supersolution. By applying [5, Theorem 22.3, chapter III], there exists a stable periodic solution $u^{*}(\xi ,t)$ to

102 $$\begin{aligned} \frac{\partial u^{*}}{\partial t} = {\mathcal {L}}u^{*} +f(u^{*}) \qquad \text {in } \xi \in \Omega _0, \ t\in {\mathbb {R}} \end{aligned}$$

103 $$\begin{aligned} u^{*}(\xi ,t)=0 \qquad \text {\qquad }\text {for } \xi \in \partial \Omega _0 \end{aligned}$$

104 $$\begin{aligned} u^{*}(\xi ,t)\equiv u^{*}(\xi ,t+T) \end{aligned}$$

such that

105 $$\begin{aligned} \varepsilon \phi (\xi ,t)e^{\alpha (t-T)} \le u^{*}(\xi ,t) \le K \qquad \text {for } 0\le t\le T,\ \xi \in \Omega _0. \end{aligned}$$

In the remainder of this section, we shall prove that the periodic solution $u^{*}$ is unique and that for any initial conditions $0\le u(\xi ,0)\le K$ not identically zero, the solution $u(\xi ,t)$ to the problem (10), (11) converges to $u^{*}$.

### Remark 8

It is straightforward to derive a lower bound on $u(\xi ,t)$ which shows that $u(\xi ,t)$ cannot converge to zero. By the strong maximum principle and Hopf’s lemma, we can assume without loss of generality that there exists $0<\delta \le \varepsilon e^{-\alpha T}$ such that $\delta \phi (\xi ,0) \le u(\xi ,0)$ (since this will hold for every time $t_0>0$). Then, since $\delta \phi (\xi ,t)e^{\alpha t}$ is a subsolution on $0\le t\le T$,

$$\begin{aligned} \delta \phi (\xi ,t')e^{\alpha t'} \le u(\xi ,t') \qquad \text {for all } 0\le t'\le T. \end{aligned}$$

Then as a consequence of the *T*-periodicity of $\phi$, $\delta \phi (\xi ,0)\le \delta \phi (\xi ,0)e^{\alpha T} \le u(\xi ,T)$ and we can conclude that

106 $$\begin{aligned} \delta \phi (\xi ,t')e^{\alpha t'} \le u(\xi ,t'+nT) \qquad \text {for all }0\le t'\le T,\ n\in {\mathbb {N}}. \end{aligned}$$

Therefore

107 $$\begin{aligned} \liminf _{t\rightarrow \infty } u(\xi ,t) \ge \delta \min _{0\le t' \le T}(\phi (\xi ,t')e^{\alpha t'}). \end{aligned}$$

To prove the convergence to $u^{*}$, we shall use the Poincaré map $P_T$. For each $\tau > 0$ define $P_{\tau }$ to be the map $P_{\tau }(u_0)=u(\cdot ,{\tau })$ where *u* is the solution to the problem (10), (11) with initial conditions $u(\cdot ,0)=u_0(\cdot )$. Since the coefficients are periodic, this is the same as the map taking $u(\cdot , nT)$ to $u(\cdot , nT+\tau )$ for any $n\in {\mathbb {N}}$. The Poincaré map is $P_T$, which takes the solution at time *nT* to the solution at time $(n+1)T$. If $u^{*}$ is a *T*-periodic solution (satisfying (102), (103), (104)), then $u^{*}(\cdot , 0)$ is a fixed point of the Poincaré map $P_T$.

We shall use the following two properties of $P_{\tau }$.

### Lemma 3

Monotonicity of $P_{\tau }$. For any $\tau >0$, the map $P_{\tau }$ is monotonic: if $u_0 \le v_0$ then $P_{\tau }(u_0) \le P_{\tau }(v_0)$. Moreover, either $u_0 \equiv v_0$ or else there is strict inequality $P_{\tau }(u_0) < P_{\tau }(v_0)$ in $\Omega _0$ and the normal derivatives satisfy $\frac{\partial }{\partial \nu }P_{\tau }(u_0) \ne \frac{\partial }{\partial \nu }P_{\tau }(v_0)$ on $\partial \Omega _0$.

### Proof

This is a consequence of the parabolic comparison principle, strong maximum principle, and Hopf’s Lemma. $\square$

### Lemma 4

Sublinearity of $P_{\tau }$. Let *f* satisfy (3). Then for any $\tau >0$, the map $P_{\tau }$ is sublinear, in the following sense. Let $0\le \alpha \le 1$, and let $u_0>0$ on $\Omega _0$, with $u_0=0$ and $\frac{\partial u_0}{\partial \nu }\ne 0$ on $\partial \Omega _0$. Then

108 $$\begin{aligned} \alpha P_{\tau }(u_0) \le P_{\tau }(\alpha u_0). \end{aligned}$$

### Proof

If $\alpha =0$ or 1 then it is obvious, so assume $0<\alpha <1$. Let $u(\xi ,t)$ be the solution to (10), (11) with initial conditions $u(\xi ,0)=u_0(\xi )$ and $v(\xi ,t)$ the solution with initial conditions $v(\xi ,0)=\alpha u_0(\xi )$. We need to show that $\alpha u(\xi ,t) \le v(\xi ,t)$ for all $t\ge 0$.

By the assumption that $\frac{f(k)}{k}$ is non-increasing, we have $f(\alpha u_0)\ge \alpha f(u_0)$. For $\varepsilon >0$ small, define $f_{\varepsilon }(k)=f(k)-\varepsilon k^2$, so that $\frac{f_{\varepsilon }(k)}{k}$ is strictly decreasing in $k>0$, and

109 $$\begin{aligned} f_{\varepsilon }(\alpha u_0)- \alpha f_{\varepsilon }(u_0) \ge \varepsilon \alpha (1-\alpha )u_0^2 \ >0\quad \text {in }\Omega _0. \end{aligned}$$

Let $v_{\varepsilon }$, $u_{\varepsilon }$ be the corresponding solutions to the problem with *f* replaced by $f_{\varepsilon }$:

110 $$\begin{aligned} \frac{\partial u_{\varepsilon }}{\partial t}={\mathcal {L}}u_{\varepsilon } +f_{\varepsilon }(u_{\varepsilon }), \qquad \frac{\partial v_{\varepsilon }}{\partial t}={\mathcal {L}}v_{\varepsilon } +f_{\varepsilon }(v_{\varepsilon }). \end{aligned}$$

We shall show that $\alpha u_{\varepsilon }(\xi ,t) \le v_{\varepsilon }(\xi ,t)$ for every $t\ge 0$. Then by taking $\varepsilon \rightarrow 0$ we conclude that the same inequality holds for the solutions *v*, *u* with the original reaction function *f*.

At $t=0$ we have $v_{\varepsilon }(\cdot ,0)- \alpha u_{\varepsilon }(\cdot ,0)=0$ and

111 $$\begin{aligned} \frac{\partial }{\partial t}(v_{\varepsilon }-\alpha u_{\varepsilon })\vert _{t=0}&= {\mathcal {L}}(\alpha u_0)+f_{\varepsilon }(\alpha u_0) - \alpha {\mathcal {L}}(u_0)-\alpha f_{\varepsilon }(u_0)\end{aligned}$$

112 $$\begin{aligned}&=f_{\varepsilon }(\alpha u_0) -\alpha f_{\varepsilon }(u_0) \ge \varepsilon \alpha (1-\alpha )u_0^2. \end{aligned}$$

Therefore, there exists $t_0>0$ such that $0\le v_{\varepsilon }(\xi ,t)- \alpha u_{\varepsilon }(\xi ,t)$ for $0\le t\le t_0$. If $t_0$ can be taken as large as we like, then we are done. Otherwise, let $t^{*}$ be the maximal such that $\alpha u_{\varepsilon }(\xi ,t) \le v_{\varepsilon }(\xi ,t)$ for $0\le t\le t^{*}$. Let ${\tilde{v}}_{\varepsilon }$ be the solution on $t\ge t^{*}$ with $\tilde{v_{\varepsilon }}(\xi ,t^{*})=\alpha u_{\varepsilon }(\xi ,t^{*})$. Then by applying the same argument as above, to the function ${\tilde{v}}_{\varepsilon }$ at time $t^{*}$, we deduce that there exists $t_1>0$ such that $\alpha u_{\varepsilon } \le {\tilde{v}}_{\varepsilon }$ for $t^{*}\le t\le t^{*}+t_1$. Since ${\tilde{v}}_{\varepsilon }\le v_{\varepsilon }$, this contradicts the maximality of $t^{*}$. Therefore, we do have $\alpha u_{\varepsilon }(\xi ,t) \le v_{\varepsilon }(\xi ,t)$ for all $t\ge 0$, as required. $\square$

### Theorem 8

Uniqueness of periodic solution (given ordering).

Suppose $f'(0)>\mu$, and suppose that ${\underline{U}}(\xi ,t)$, ${\overline{U}}(\xi ,t)$ are both positive, *T*-periodic solutions to the problem (102), (103), (104) with $0\le {\underline{U}}(\xi ,0)\le {\overline{U}}(\xi ,0)$ for all $\xi \in \Omega _0$. Then ${\underline{U}}(\xi ,t)\equiv {\overline{U}}(\xi ,t)$.

### Proof

By the strong maximum principle and Hopf’s lemma, $0<{\underline{U}}(\xi ,0)\le {\overline{U}}(\xi ,0)$ for all $\xi \in \Omega _0$, and ${\underline{U}}$ and ${\overline{U}}$ have non-zero normal derivatives on $\partial \Omega _0$. Therefore for $r>0$ small enough we have $r{\overline{U}}(\xi ,0)\le {\underline{U}}(\xi ,0)$ for all $\xi \in \Omega _0$. On the other hand this does not hold for any $r>1$. Let

113 $$\begin{aligned} {\hat{r}}=\sup \{ r\in (0,1): r{\overline{U}}(\xi ,0)\le {\underline{U}}(\xi ,0) \text { for all } \xi \in \Omega _0 \}. \end{aligned}$$

Then we know that

114 $$\begin{aligned} {\hat{r}}{\overline{U}}(\xi ,0)\le {\underline{U}}(\xi ,0) \text { for all } \xi \in \Omega _0 \end{aligned}$$

and (by maximality of ${\hat{r}}$) there exists some

115 $$\begin{aligned}&\xi _0\in \Omega _0 \text { such that }{\hat{r}}{\overline{U}}(\xi _0,0) = {\underline{U}}(\xi _0,0) \nonumber \\&\text {or}\qquad \xi _0 \in \partial \Omega _0 \text { such that } {\hat{r}}\frac{\partial {\overline{U}}}{\partial \nu }(\xi _0,0) = \frac{\partial {\underline{U}}}{\partial \nu }(\xi _0,0). \end{aligned}$$

Now apply the Poincaré map, $P_T$. By the monotonicity (Lemma 3) we have

116 $$\begin{aligned} P_T({\hat{r}}{\overline{U}}(\cdot ,0)) \le P_T({\underline{U}}(\cdot ,0)) \end{aligned}$$

with either ${\hat{r}}{\overline{U}} \equiv {\underline{U}}$ or else strict inequality

117 $$\begin{aligned} P_T({\hat{r}}{\overline{U}}(\cdot ,0)) < P_T({\underline{U}}(\cdot ,0)) \qquad \text {on } \Omega _0 \end{aligned}$$

and

118 $$\begin{aligned} \frac{\partial }{\partial \nu }P_T({\hat{r}}{\overline{U}}(\cdot ,0)) \ne \frac{\partial }{\partial \nu }P_T({\underline{U}}(\cdot ,0)) \qquad \text {on } \partial \Omega _0. \end{aligned}$$

Combining this with the sublinearity property (Lemma 4) and the fact that ${\overline{U}}$ and ${\underline{U}}$ are fixed points of $P_T$, we find that

119 $$\begin{aligned} {\hat{r}}{\overline{U}}(\cdot ,0) = {\hat{r}}P_T({\overline{U}}(\cdot ,0)) \le P_T({\hat{r}}{\overline{U}}(\cdot ,0)) \le P_T({\underline{U}}(\cdot ,0))={\underline{U}}(\cdot ,0) \end{aligned}$$

and that either ${\hat{r}}{\overline{U}} \equiv {\underline{U}}$ or else Eqs. (117) and (118) hold. Incorporating these strict inequalities into Eq. (119) would contradict the existence of $\xi _0$ as in Eq. (115). Therefore, in fact

120 $$\begin{aligned} {\hat{r}}{\overline{U}} \equiv {\underline{U}} \qquad \text {on } \overline{\Omega _0}\times [0,T]. \end{aligned}$$

This shows that ${\overline{U}}$ and ${\hat{r}}{\overline{U}}$ are both solutions to (102), (103), (104), and hence ${\hat{r}}f({\overline{U}})\equiv f({\hat{r}}{\overline{U}})$. By the assumption that $\frac{f(k)}{k}$ is non-increasing on $k>0$, this implies that either ${\hat{r}}=1$ or else $f({\overline{U}}) \equiv f'(0){\overline{U}}$. But we know that ${\overline{U}}$ does not satisfy the linear equation because that would contradict the fact that $f'(0)>\mu$. Therefore, it must be that ${\hat{r}}=1$ and ${\underline{U}}\equiv {\overline{U}}$. $\square$

Now we are able to prove convergence to $u^{*}(\xi ,t)$ (the positive *T*-periodic solution to (102), (103), (104) whose existence is guaranteed by [5, Theorem 22.3, chapter III]).

### Theorem 9

Assume that *f* satisfies (3) and $f'(0)>\mu$, and let $u^{*}(\xi ,t)$ be a positive *T*-periodic solution to (102), (103), (104). Given non-negative, not identically zero initial conditions $0\le u(\xi ,0)\le K$, let $u(\xi ,t)$ be the solution to the nonlinear problem (10), (11), and for $n\in {\mathbb {N}}$ define $u_n(\xi , t)=u(\xi ,nT+t)$. Then as $n\rightarrow \infty$, $u_n$ converges in $C^{2,1}(\overline{\Omega _0}\times [0,T])$ to $u^{*}(\xi ,t)$. In particular, $u^{*}$ is unique.

### Proof

Without loss of generality (since it will hold for every time $t_0>0$ by the strong maximum principle and Hopf’s Lemma) we can assume that the initial conditions are such that

121 $$\begin{aligned} \delta u^{*}(\xi ,0) \le u(\xi , 0)\le Bu^{*}(\xi ,0) \end{aligned}$$

for some $0<\delta \le 1$ and $B\ge 1$. Let ${\underline{u}}(\xi ,t)$ and ${\overline{u}}(\xi ,t)$ be the solutions to (10), (11) with initial conditions ${\underline{u}}(\xi ,0)=\delta u^{*}(\xi ,0)$ and ${\overline{u}}(\xi ,0)=B u^{*}(\xi ,0)$. By the comparison principle,

122 $$\begin{aligned} {\underline{u}}(\cdot ,t)\le u(\cdot ,t)\le {\overline{u}}(\cdot ,t) \qquad \text {and}\qquad {\underline{u}}(\cdot ,t)\le u^{*}(\cdot ,t)\le {\overline{u}}(\cdot ,t)\qquad \text {for all } t\ge 0. \end{aligned}$$

For $n\in {\mathbb {N}}$ define $u_n(\xi , t)=u(\xi ,nT+t)$; also define ${\underline{u}}_n(\xi ,t)={\underline{u}}(\xi ,nT+t)$ and ${\overline{u}}_n(\xi ,t)={\overline{u}}(\xi ,nT+t)$. Then we have

123 $$\begin{aligned} {\underline{u}}_n(\cdot ,t)\le u_n(\cdot ,t)\le {\overline{u}}_n(\cdot ,t)\qquad \text {and}\qquad {\underline{u}}_n(\cdot ,t)\le u^{*}(\cdot ,t)\le {\overline{u}}_n(\cdot ,t) \end{aligned}$$

for all $$0\le t\le T$$, $$n\in {\mathbb {N}}$$.

Using the fact that $u^{*}(\cdot , 0)$ is a fixed point of the Poincaré map $P_T$, together with the sublinearity of $P_T$ (Lemma 4), we get that for all $\xi \in \Omega _0$,

124 $$\begin{aligned} {\underline{u}}(\xi ,0)=\delta u^{*}(\xi ,0) = \delta P_T(u^{*}(\xi ,0)) \le P_T(\delta u^{*}(\xi ,0))= P_T({\underline{u}}(\xi ,0))={\underline{u}}(\xi ,T) \end{aligned}$$

and

125 $$\begin{aligned} {\overline{u}}(\xi ,0)= Bu^{*}(\xi ,0) =B P_T(u^{*}(\xi ,0))&= BP_T\left( \frac{1}{B} \ Bu^{*}(\xi ,0)\right) \nonumber \\&\ge P_T( Bu^{*}(\xi ,0))= P_T({\overline{u}}(\xi ,0))= {\overline{u}}(\xi ,T). \end{aligned}$$

Therefore, ${\underline{u}}(\xi ,0) \le {\underline{u}}(\xi ,T)$ and ${\overline{u}}(\xi ,T) \le {\overline{u}}(\xi ,0)$. Apply $P_T$ again and use the monotonicity property (Lemma 3) and the ordering (122), to deduce that

126 $$\begin{aligned} {\underline{u}}(\xi , nT)\le {\underline{u}}(\xi , (n+1)T) \le u^{*}(\xi , 0)\le {\overline{u}}(\xi , (n+1)T)\le {\overline{u}}(\xi , nT) \end{aligned}$$

for all $\xi \in \Omega _0$, $n\in {\mathbb {N}}$. Therefore, pointwise limits ${\underline{v}}(\xi )$ and ${\overline{v}}(\xi )$ exist such that ${\underline{v}}(\xi ) \le u^{*}(\xi , 0)\le {\overline{v}}(\xi )$ and

127 $$\begin{aligned} {\underline{u}}(\xi , nT)\rightarrow {\underline{v}}(\xi ), \qquad {\overline{u}}(\xi ,nT)\rightarrow {\overline{v}}(\xi ) \qquad \text {as }n\rightarrow \infty . \end{aligned}$$

Using parabolic estimates from [8, Lemma 7.20 and Theorem 7.30] and [7, chapter IV, Theorem 10.1] and embeddings from [6, Theorem 3.14(3)], we deduce that there is a subsequence ${\underline{u}}_{n_k}$ which converges in $C^{2,1}(\overline{\Omega _0}\times [0,T])$ to a solution ${\underline{U}}(\xi ,t)$ of the nonlinear parabolic problem (10), (11). By equating this to the pointwise limit at times 0 and *T*, we have that ${\underline{U}}(\xi ,0)={\underline{U}}(\xi ,T)={\underline{v}}(\xi )$. Likewise, there is a subsequence ${\overline{u}}_{n_r}$ of ${\overline{u}}_n$ which converges in $C^{2,1}(\overline{\Omega _0}\times [0,T])$ to a solution ${\overline{U}}(\xi ,t)$ of (10), (11), with ${\overline{U}}(\xi ,0)={\overline{U}}(\xi ,T)={\overline{v}}(\xi )$.

Now ${\underline{U}}(\xi ,0)={\underline{v}}(\xi )\le u^{*}(\xi , 0)\le {\overline{v}}(\xi )={\overline{U}}(\xi ,0)$ and so by the comparison principle, ${\underline{U}}(\xi ,t)\le u^{*}(\xi , t)\le {\overline{U}}(\xi ,t)$ for all $t\ge 0$. Therefore ${\underline{U}}$ and ${\overline{U}}$ satisfy the conditions of Theorem 8, and we conclude that

128 $$\begin{aligned} {\underline{U}}\equiv {\overline{U}} \equiv u^{*}. \end{aligned}$$

Since the limit is uniquely identified, this implies that actually the whole sequences ${\underline{u}}_n$ and ${\overline{u}}_n$ converge to $u^{*}$ as $n\rightarrow \infty$ and the convergence is in $C^{2,1}(\overline{\Omega _0}\times [0,T])$. But since $u_n$ satisfies (123), it must also converge uniformly to $u^{*}$ as $n\rightarrow \infty$, and by the same argument as above the convergence is in $C^{2,1}(\overline{\Omega _0}\times [0,T])$. $\square$

The convergence of $u(\xi ,nT+t)$ to a unique positive *T*-periodic solution $u^{*}(\xi ,t)$ on $\Omega _0\times [0,T]$ can now be interpreted in terms of the original problem for $\psi (x,t)$ on the *T*-periodic domain $\Omega (t)$. The function $u^{*}(\xi ,t)$ for $\xi \in \Omega _0$ corresponds to a positive solution $\psi ^{*}(x,t)$ to (1), (2) such that $\psi ^{*}(x,t)\equiv \psi ^{*}(x,t+T)$ for all $x\in \Omega (t)$, $t\in {\mathbb {R}}$. Theorem 9 means that $\psi (x,nT+t)$ converges uniformly to $\psi ^{*}(x,t)$ as $n\rightarrow \infty$.
